# Characterization of the first tetrameric transcription factor of the GntR superfamily with allosteric regulation from the bacterial pathogen *Agrobacterium fabrum*

**DOI:** 10.1093/nar/gkaa1181

**Published:** 2020-12-11

**Authors:** Armelle Vigouroux, Thibault Meyer, Anaïs Naretto, Pierre Legrand, Magali Aumont-Nicaise, Aurélie Di Cicco, Sébastien Renoud, Jeanne Doré, Daniel Lévy, Ludovic Vial, Céline Lavire, Solange Moréra

**Affiliations:** Université Paris-Saclay, CEA, CNRS, Institute for Integrative Biology of the Cell (I2BC), 91198 Gif-sur-Yvette, France; Univ Lyon, Université Claude Bernard Lyon 1, CNRS, INRAE, VetAgro Sup, UMR Ecologie Microbienne, F-69622 Villeurbanne, France; Université Paris-Saclay, CEA, CNRS, Institute for Integrative Biology of the Cell (I2BC), 91198 Gif-sur-Yvette, France; Synchrotron SOLEIL, L’Orme des Merisiers, Saint-Aubin, 91192 Gif-sur-Yvette, France; Université Paris-Saclay, CEA, CNRS, Institute for Integrative Biology of the Cell (I2BC), 91198 Gif-sur-Yvette, France; Sorbonne Université, Laboratoire Physico Chimie Curie, Institut Curie, PSL Research University, CNRS UMR168, 26 rue d’Ulm, 75005 Paris, France; Univ Lyon, Université Claude Bernard Lyon 1, CNRS, INRAE, VetAgro Sup, UMR Ecologie Microbienne, F-69622 Villeurbanne, France; Univ Lyon, Université Claude Bernard Lyon 1, CNRS, INRAE, VetAgro Sup, UMR Ecologie Microbienne, F-69622 Villeurbanne, France; Sorbonne Université, Laboratoire Physico Chimie Curie, Institut Curie, PSL Research University, CNRS UMR168, 26 rue d’Ulm, 75005 Paris, France; Univ Lyon, Université Claude Bernard Lyon 1, CNRS, INRAE, VetAgro Sup, UMR Ecologie Microbienne, F-69622 Villeurbanne, France; Univ Lyon, Université Claude Bernard Lyon 1, CNRS, INRAE, VetAgro Sup, UMR Ecologie Microbienne, F-69622 Villeurbanne, France; Université Paris-Saclay, CEA, CNRS, Institute for Integrative Biology of the Cell (I2BC), 91198 Gif-sur-Yvette, France

## Abstract

A species-specific region, denoted SpG8-1b allowing hydroxycinnamic acids (HCAs) degradation is important for the transition between the two lifestyles (rhizospheric versus pathogenic) of the plant pathogen *Agrobacterium fabrum*. Indeed, HCAs can be either used as trophic resources and/or as induced-virulence molecules. The SpG8-1b region is regulated by two transcriptional regulators, namely, HcaR (Atu1422) and Atu1419. In contrast to HcaR, Atu1419 remains so far uncharacterized. The high-resolution crystal structures of two fortuitous citrate complexes, two DNA complexes and the apoform revealed that the tetrameric Atu1419 transcriptional regulator belongs to the VanR group of Pfam PF07729 subfamily of the large GntR superfamily. Until now, GntR regulators were described as dimers. Here, we showed that Atu1419 represses three genes of the HCAs catabolic pathway. We characterized both the effector and DNA binding sites and identified key nucleotides in the target palindrome. From promoter activity measurement using defective gene mutants, structural analysis and gel-shift assays, we propose N5,N10-methylenetetrahydrofolate as the effector molecule, which is not a direct product/substrate of the HCA degradation pathway. The Zn*^2+^* ion present in the effector domain has both a structural and regulatory role. Overall, our work shed light on the allosteric mechanism of transcription employed by this GntR repressor.

## INTRODUCTION

**Figure 1.**
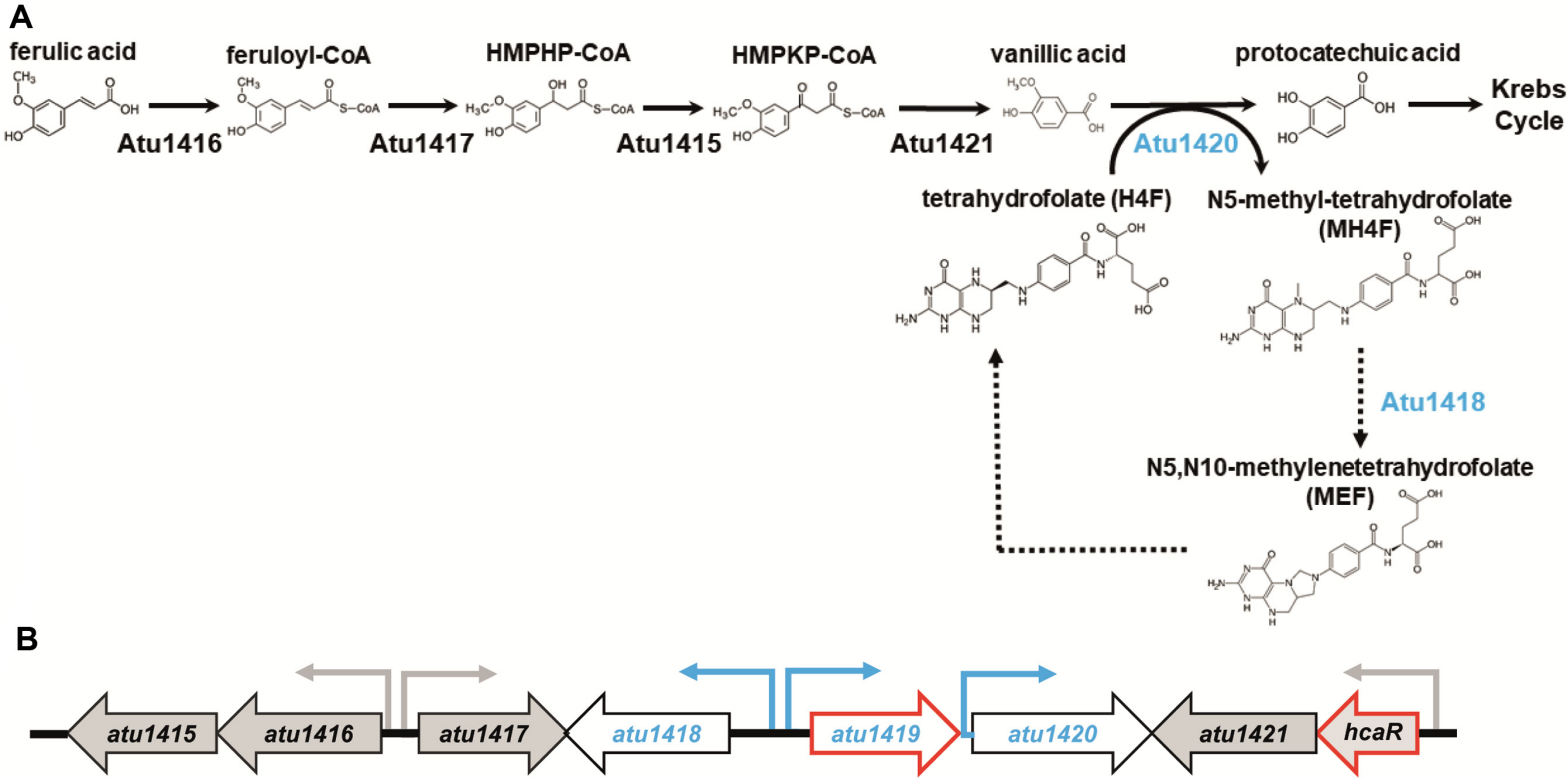
(**A**) Ferulic acid degradation pathway: a coenzyme A is added to ferulic acid by Atu1416, a feruloyl-CoA synthase. Feruloyl-CoA is then converted by Atu1417, an enoyl-CoA hydratase, into 4-hydroxy-3-methoxyphenyl-β-hydroxypropionyl (HMPHP)-CoA, which is in turn transformed into vanillic acid by Atu1415, a phenylhydroxypropionyl-CoA dehydrogenase, and then Atu1421, a 4-hydroxy-3-methoxyphenyl-β-ketopropionyl-CoA (HMPKP)-CoA β-keto-thiolase. The O-demethylase Atu1420 degrades vanillic acid into protocatechuic acid using tetrahydrofolate (H4F) as a cofactor and produces N5-methyl-tetrahydrofolate (MH4F). Protocatechuic acid enters the cycle of Krebs. Atu1418 enzyme was proposed to be involved in the recycling of H4F from transformation of MH4F into MEF, a compound that can be enzymatically or spontaneously converted to H4F (18,50). Dashed arrows indicated putative reactions based on sequence similarities and references (4,18,44,50). (**B**) Organization of the SpG8–1b region for which genes expression is regulated by the two transcriptional factors HcaR and Atu1419 (their genes are framed in red). Promoters are shown with small arrows in black for HcaR dependent-transcription and in blue for putative Atu1419 dependent-transcription indicating the direction of gene transcription.

*Agrobacterium fabrum* has two lifestyles: it can interact with a large variety of plants as a rhizosphere inhabitant or as a pathogen when it harbors a tumor-inducing plasmid (which is a virulence plasmid), and transfers a portion of this to the plant cells upon infection, resulting in the crown-gall disease (1–3). *A. fabrum* possesses a species-specific region, denoted SpG8-1b (a region present in strains of this species but absent from other *Agrobacterium* species), located in the circular chromosome and responsible for hydroxycinnamic acids (HCAs) degradation such as ferulic acid, caffeic acid and *p*-coumaric acid (4,5) (Figure 1). These latter compounds are common plant secondary metabolites being precursors of lignin incorporated into plant cell walls. They are abundantly released in soil during the decay of root cells and are significant environmental molecules for soil- and plant-interacting bacteria (6). Although HCAs are generally a strong bacterial repellent, they appear to be chemoattractants in the case of rhizobia and agrobacteria for which they can be used as trophic resources and/or induced-virulence molecules (7–11). We have previously showed that HCAs degradation *via* the SpG8-1b region interferes with virulence gene expression suggesting that this metabolic pathway is important for the transition between the two lifestyles (rhizospheric versus pathogenic) of *Agrobacterium* (11). Such a transition requires fine-tuning of the regulation of gene expression to express the appropriate genes at the right time (12–14).

The SpG8-1b genomic region encodes six enzymes (Figure 1A). Five of them operate in this sequential order for the degradation of ferulic acid leading to different intermediates (5): Atu1416 (a feruloyl-CoA synthase), Atu1417 (an enoyl-CoA hydratase), Atu1415 (a phenylhydroxypropionyl-CoA dehydrogenase), Atu1421 (a 4-hydroxy-3-methoxyphenyl-β-ketopropionyl-CoA β-keto-thiolase) and Atu1420 (a tetrahydrofolate-dependent vanillate O-demethylase). Atu1420 shares 56% sequence identity with *Sphingomonas paucimobilis* and *Sphingobium* sp. SYK-6 homologues of known structures (PDB 5TL4 (15) and PDB 5X1I (16), respectively). This enzyme degrades vanillic acid into protocatechuic acid using the tetrahydrofolate (H4F) cofactor and producing the N5-methyl-tetrahydrofolate compound (MH4F) (5). Protocatechuic acid is then processed by *pca* genes to produce intermediates of the tricarboxylic acid (TCA) cycle (17). Based on sequence identity of 29% with the N5,N10-methylene tetrahydrofolate reductase MetF of *Sphingobium* sp. SYK-6, Atu1418 the sixth enzyme of the SpG8-1b genomic region, is likely a 5,10-methylene tetrahydrofolate reductase which transforms MH4F into N5,N10-methylenetetrahydrofolate (MEF), which would, in turn, allow the regeneration of H4F (4,18) (Figure 1A).

Although the SpG8-1b genomic region degrades both ferulic and *p*-coumaric acids, this latter induces the gene expression of the first part of the SpG8-1b region while ferulic acid induces that of the whole pathway (11). Therefore, two expression units are differently regulated suggesting the existence of two regulators. These regulators are Atu1419 (predicted to be a GntR member) and Atu1422 denoted HcaR (for hydroxycinnamic acid catabolic repressor belonging to the MarR family), respectively (4,11) (Figure 1B). HcaR is the repressor of its own transcription and that of the first *atu1416* and *atu1417* genes of HCA degradation pathway (11). However, because *atu1415* and *atu1416* belong to the same transcription unit as well as *atu1421* and *hcaR* do, HcaR also regulates *atu1415* and *atu1421* genes expression, completing the regulation of five genes (Figure 1B). In contrast to HcaR, the second regulatory protein Atu1419 likely involved in the second part of the pathway corresponding to the vanillate degradation remains uncharacterized so far.

Herein, we investigated the molecular role and structural aspects of Atu1419 combining *in vitro* and *in vivo* approaches. We first proved that Atu1419 was a transcription repressor of three genes of the second part of the HCA degradation pathway. None of HCA degradation intermediates could release Atu1419 from DNA binding. Nonetheless, structural analysis of five high-resolution crystal structures of Atu1419 in apoform, in complex with a fortuitous citrate molecule bound to the effector domain originated from the crystallization condition (two structures), in complex with DNA and in complex with both DNA and citrate, helped us infer a possible effector molecule, which was confirmed by gel-shift assays and microcalorimetry. The structures revealed Atu1419 to be a member of the VanR group of the FadR C-terminal domain (FCD; Pfam PF07729) subfamily of the large GntR superfamily of transcriptional factors (>93 135 members in Pfam database) (19–22). The FCD subfamily encompasses two groups of regulators namely FadR and VanR. So far, the dimeric FadR from *Escherichia coli* was the best characterized of the FadR group of FCD-GntR regulators shown to be regulated by the acyl-CoA effector and able to bind specific palindromic DNA through a winged Helix-Turn-Helix (wHTH) motif (23). Like FadR, Atu1419 possesses a characteristic molecular architecture, composed of a conserved N-terminal DNA binding domain containing the wHTH motif and a C-terminal effector binding/oligomerization domain. Unlike FadR, this latter domain of Atu1419 displays six helices instead of seven observed in members of the FadR group. Atu1419 is the first example of a transcriptional regulator of the whole GntR superfamily to be tetrameric. Our work brings new insights into mechanistic aspects of such repressor, which uses an induced-allosteric mechanism for DNA release upon effector binding.

## MATERIALS AND METHODS

### Bacterial strains and growth conditions

The bacteria and plasmids used for this study are listed in Supplementary Table S1. *Escherichia coli* were grown routinely, with shaking (150 rpm), at 37°C in LB medium. Growth media were supplemented with appropriate antibiotics (tetracycline, 10 μg/ml; gentamicin, 15 μg/ml; ampicillin, 100 μg/ml) when necessary. The *A. fabrum* strains were grown with shaking (160 rpm), at 28°C in YPG (Yeast Peptone Glucose)-rich medium or in AT minimal medium supplemented with 10 mM succinate and 10 mM ammonium sulfate. AT minimal medium was supplemented with 750 μM of ferulic acid or citrate and with the appropriate antibiotic (gentamicin, 10 μg/ml). Ferulic acid and MH4F were obtained from Sigma Aldrich (St. Louis, USA) and MEF from Merck Company (Switzerland).

### Construction of the deletion mutant C58Δ*atu1419* and transcriptional fusions

The C58Δ*atu1419* and C58Δ*atu1420* strains were constructed according to a strategy as described (4,11). Vectors containing the recombinant region (amplified by PCR with specific primers listed in Supplementary Table S2), flanking downstream and upstream of the *atu1419* or *atu1420* genes, were introduced into *A. fabrum* C58 by electroporation. Single-crossover integration was selected by gentamycin resistance on YPG medium plates. Gentamycin-resistant colonies were spread on YPG plates containing 5% of sucrose to obtain plasmid excision and double-crossover events leading to nonpolar mutants. *atu1419* and *atu1420* deletions were confirmed by PCR analysis and DNA sequencing (GenoScreen, Lille, France).

pOT1e (24) transcriptional fusions of the promoter regions of SpG8-1b genes, namely P*atu1419* and P*atu1420* were generated as described (11) (Supplementary Table S2 for specific primers). Reporter constructions were introduced into *A. fabrum* C58 wild-type and derivatives by electroporation, and gentamycin-resistant colonies were selected.

### Measurement of promoter activity

Genes expression was measured after 24 h in *A. fabrum* with a pOT1e plasmid harboring an eGFP transcriptional fusion (24) as described (11). Results were normalized by dividing the fluorescence level by the optical density at 600 nm values. At least five technical replicates and two biological replicates were performed for each condition. Differences between conditions were determined with Tukey test (*P*-value = 0.05).

### Oligonucleotides and DNA preparation

The synthetic palindromic oligodeoxyribonucleotide 5′-ATGTATACAT-3′ was purchased from Sigma-Aldrich (Darmstadt, Germany). Oligonucleotide solution in sterile water at 2 mM was hybridized by heating to 90°C for 5 min and cooling in the crystallization room at 18°C overnight to produce a 10-mer DNA.

The *atu1420* promoter regions were amplified with specific primer pairs listed in Supplementary Table S2. The *atu1416–1417*, *atu1418–1419*, *virB* and *hcaR* promoter regions were prepared as previously described (11) and purified with a PCR Clean-up kit (Macherey-Nagel, Düren, Germany).

### Cloning, expression and purification of Atu1419 and Atu1419-H3A mutant

Coding sequences for Atu1419 was amplified by PCR adding a C-terminal 6-Histidine tag and using *atu1419F* and *atu1419R* primers and was inserted into the NdeI/XhoI restriction sites of the pET-20b vector (Novagen, Merck Biosciences, France). The nucleotide sequence was confirmed by DNA-sequence analysis (GATC Biotech, Mulhouse, France). *E. coli* BL21 competent cells transformed with pET-20b-Atu1419 were grown in LB media until OD_600_ of 0.8 and protein production was induced by 0.5 mM isopropyl β-d-thio-galactopyranoside (IPTG) for 3 h at 37°C. Cells were centrifuged at 4000 g for 15 min at 4°C, resuspended in a buffer of 50 mM Tris–HCl pH 8, 300 mM NaCl and 20 mM imidazole and disrupted by sonication. After centrifugation at 25 000 g for 30 min at 4°C, the filtrated supernatant was loaded onto a 5 ml His-Trap HP column (GE Healthcare, Chicago, IL, USA). After a washing step of 6% with 50 mM Tris–HCl pH 8, 300 mM NaCl and 300 mM imidazole (Buffer B), protein elution was performed with Buffer B. Protein fractions were loaded onto a gel filtration column (HiLoad 26/60 Superdex 200 prep grade, GE Healthcare) equilibrated with 50 mM Tris–HCl pH 8 and 150 mM NaCl. The protein fractions were pooled, concentrated and stored at −80°C.

The synthetic gene (Genscript, Piscataway, NJ, USA) coding for Atu1419 mutant (H192A, H141A and H214A) namely Atu1419-H3A was inserted into pET-20b. The Atu1419-H3A mutant was expressed and purified as the wild-type protein described above.

#### Size exclusion chromatography (SEC) and SEC-MALS

For size exclusion chromatography (SEC) and SEC coupled to multi-angle light scattering (SEC-MALS) analyses, apo Atu1419 was prepared at 25 μM and Atu1419 in complex with the palindromic DNA was prepared with a ratio protein:DNA of 1:2 in a buffer containing 50 mM Tris pH 8 and 150 mM NaCl. The same buffer was used as the mobile phase for SEC using a Superdex 200 10/300 GL column on an AKTA FPLC system (GE Healthcare) and on a Shimadzu HPLC. Multiangle light scattering was detected with a MiniDAWN TREOS light scattering module and a refractometer Optilab T-rEX (Wyatt Technology).

### Electrophoretic mobility shift assay (EMSA)/Gel-shift assay

Atu1419 or Atu1419-H3A mutant were mixed with different promoter regions (P*atu1416–1417*, P*atu1418–1419*, P*hcaR*, P*atu1420* and P*virB*) and with variants of the *atu1418–1419* and *atu1420* regions. The intergenic regions of *virB* was used as a nonspecific control probe. Gel-shift assays/EMSA were performed in 10 μl reaction mixture containing 30 nM of DNA probe without and with Atu1419 at different concentrations in 50 mM Tris–HCl pH 8 and 150 mM NaCl. 50 to 300 μM of MEF, MH4F, H4F or citrate were added for testing their influence on Atu1419–DNA complex formation. After incubation for 30 min at room temperature, the samples were separated by electrophoresis in TBE buffer (45 mM Tris–HCl pH 8, 45 mM boric acid and 1 mM EDTA) on non-denaturing 6% or 12% polyacrylamide gels at 150 V and 4°C for 2 h. Gels were then stained with either SYBR® Green EMSA nucleic acid gel stain (Invitrogen, Carlsbad, CA, USA) or ethidium bromide for 20 min. DNA was visualized under UV light (Fisher Bioblock Scientific, Illkirch, France or UVP BioDoc-it2 Imager, Analytic Jena, Germany).

### Crystallization and structure determination of Atu1419

Crystallization conditions are summarized in Table 1. For all protein samples (140 μM tetrameric protein alone or in complex with 700 μM palindromic DNA), conditions were screened using QIAGEN kits (Valencia, CA, USA) with a Mosquito nanodrop robot (TTP Labtech, Melbourn, Great Britain) and were manually optimized at 20°C in hanging drop by mixing equal volumes of the protein or protein–DNA solution with precipitant solution. Crystals were transferred to a cryoprotectant solution (paraffin oil or mother liquor supplemented with 20% PEG 400) and flash-frozen in liquid nitrogen. Diffraction data were collected at 100 K on PROXIMA 1 and PROXIMA 2 beamlines at synchrotron SOLEIL (Saint-Aubin, France). Intensities were integrated using the XDS package (25) (Table 1).

**Table 1.** Crystallographic data and refinement parameters

	Zn^2+^ SAD	Atu1419 citrate	Atu1419 citrate	Atu1419-DNA#	Atu1419-DNA§	Apo Atu1419†
PDB code		6Z74	6ZA0	6ZAB	6ZA3	6ZA7
Wavelength (Å)	1.282290	0.97541	0.978570	1	1	0.97934
Crystallization conditions	5% PEG 4K, 0.2 M AS, 0.1 M Na-citrate pH 5.6	5% PEG 4K, 0.2 M AS, 0.1 M Na-citrate pH 5.6	5% PEG 4K, 0.2 M AS, 0.1 M Na-citrate pH 5.6	10% Terbutanol, 0.1 M Na-citrate pH 5.6, 2 mM MgCl_2_	25% PEG 400, 0.1 M MES pH 6.7, 0.2 M Na-acetate	12% PEG 4K, 0.2 Ammonium sulfate, 0.1 M Tris/HCl pH 8.5
*Za*	4	4	2	1	2	4
Space group Cell parameters (Å,°)	*P2_1_2_1_2_1_*	*P2_1_2_1_2_1_*	*P2_1_2_1_2*	*P6_4_22*	*C222_1_*	*P2_1_*
*a* = 77.4	*a* = 77.2	*a =* 72.2	*a* = 179.5	*a* = 62.4	*a* = 52.1
	*b* = 114,1	*b* = 113.7	*b* = 145.6	*b* = 179.2	*b* = 112.4	*b* = 79.0
	*c* = 130.5	*c* = 130.5	*c* = 41.2	*c* = 96.8	*c* = 179.6	*c* = 140.6
						β = 95.1
Resolution (Å)	49.8–2.35 (2.42–2.35)	45.6–2 (2.12–2)	24.0–1.65 (1.69–1.65)	45.61–2.8 (2.87–2.8)	47.64–2.0 (2.05–2.0)	43.38–2.34 (2.41–2.34)
No. of observed reflections	518 098 (29677)	467 551 (72154)	239 555 (17376)	444 041 (30119)	376 669 (23170)	504 810 (31752)
No. of unique reflections	48 018 (3262)	78 344 (12280)	51 917 (3674)	23 089 (1642)	43 067 (3095)	47 943 (3255)
Completeness (%)	99.4 (92.1)	99.6 (98.1)	97.9 (95.8)	99.7 (88.4)	99.9 (99.1)	99.4 (92.3)
Completeness staraniso (%)				*68.7 (11.9)*	*97.7 (79.3)*	*71.5 (30.6)*
*R_sym_* (%)	8.8 (78.6)	12 (155.5)	8.1 (127.3)	*11.6 (236.4)*	*8.6 (190.3)*	*10.9 (128.8)*
*R_pim_* (%)	2.8 (26.6)	5.4 (78.1)	4.2 (64.4)	*2.7 (57.7)*	*3.1 (79.3)*	*3.5 (41.5)*
*I*/σ*(I)*	18 (2.3)	10 (1.12)	10.7 (1.1)	*18.8 (1.1)*	*13.7 (0.9)*	*13.0 (1.4)*
CC_1/2_	0.999 (0.809)	0.998 (0.582)	0.999 (0.461)	*0.999 (0.469)*	*0.999 (0.421)*	*0.999 (0.696)*
*R_cryst_* (%)		19.1	16.9	18.8	19.2	19.4
*R_free_* (%)		21.4	18.9	19.9	22.8	22.2
rms bond deviation (Å)		0.01	0.001	0.009	0.01	0.009
rms angle deviation (°)		0.99	0.95	1	1	1.02
Average B (Å^2^)						
Protein A/B/C/D		42/53.1/60/57.5	27.9/29.2	86.4	50.7/50.1	66.2/74.3/73.2/80.8
Zn^2+^		35/41.4/39.7/45.2	18.3/18.6	106.9	43.3/46.2	46.2/63.2/69.3/66.6
Citrate		44.4/45.2/44.5/45.7	23.1/22	127.9		
DNA				65.3	60.2	
Solvent		50.5	36.7	56.3	48.0	50.0
^a^Clashscore		4.56	1.6	3.2	2.11	2.88
MolProbity score		1.51	0.93	2.07	1.24	1.38
^a^Ramachandran plot (%)						
Favoured		99.36	98.73	94.94	98.73	98.2
Outliers		0	0	0	0	0.21

Values for the highest resolution shell are in parentheses. CC_1/2_ = percentage of correlation between intensities from random hall-dataset.

^a^Calculated with MolProbity.

Numbers in italic account for statistical values after ellipsoidal mask application by Staraniso.

#A dataset collected from a crystal, which diffracted anisotropically to 3.4 Å along b* and 0.894a* – 0.447b* and 2.7 Å along c*.

†Two datasets collected from a crystal, which diffracted anisotropically to 3.2 Å along 0.81a* – 0.58c*, 2.3 Å along b* and 2.2 Å along c*.

§One dataset collected from a crystal, which diffracted anisotropically to 2.02 Å along a*, 1.99 Å along b* and 1.95 Å along c*.

The first structure of Atu1419 in *P2_1_2_1_2_1_* space group was determined at 2.35 Å resolution by single-wavelength anomalous dispersion (SAD) method at the peak absorption energy of Zinc (Table 1). The presence of a zinc metal in crystals was found thanks to an X-ray fluorescence emission scan on the beamline. The steps of zinc ion substructure determination, phases calculation and density modification were performed using CRANK from CCP4 (Collaborative Computational Project, Number 4) and a partial model was built using BUCCANEER (CCP4). This model was then used to calculate the phases of a higher resolution dataset at 2 Å resolution leading to the complete polypeptide chain model. A tetramer is present in the asymmetric unit. Other structure determinations were performed by molecular replacement with PHASER (26) using the first refined structure of Atu1419 (monomer, dimer or tetramer). Because of the anisotropy of the diffraction of Atu1419–DNA complex in *P6_4_22* crystals and apo Atu1419, the DEBYE and STARANISO programs developed by Global phasing Ltd were applied to the data scaled with XDS using the STARANISO server (http://staraniso.globalphasing.or/). These programs perform an anisotropic cut-off of merge intensity data on the basis of an analysis of local I/σ(I), compute Bayesian estimates of structures amplitudes, taking into account their anisotropic fall-off, and apply an anisotropic correction to the data. The corrected anisotropic amplitudes were used for further refinement. Refinement of each structure was performed with BUSTER-2.10 (27) employing TLS groups and NCS restraints. Inspection of the density maps and manual rebuilding were performed using COOT (28). Refinement details of each structure are shown in Table 1. Molecular graphics images were generated using PyMOL (http://www.pymol.org).

### Circular dichroism experiments (CD)

Circular dichroïsm in the far-UV region was performed using a spectropolarimeter (Jasco J-810, Jasco, Lisses, France) equipped with a water-cooled Peltier unit (Jasco circular dichroïsm spectrometer model J810). Apo Atu1419 and Atu1419-H3A mutant were both concentrated at 25 μM in 50 mM Tris pH 8 and 150 mM NaCl. Spectra were recorded in a cell width of 0.1 mm path length (121.QS, Hellma, Hellma Analytics, Müllheim, Baden Württemberg, Germany) from 190 to 240 nm at 20°C. Five consecutive scans from each sample were merged to produce an averaged spectrum; the spectra were corrected using buffer baselines measured under the same conditions. Data were recorded in mdeg and converted using the mean residues ellipticity method (deg cm^2^ mol^−1^). Secondary structure estimates were derived from the normalized spectra using the CDSSTR, SELCON3, CONTIN of the DICHROWEB server (29,30).

### Differential scanning calorimetry (auto PEAQ DSC)

Thermal stability of 10 μM apo Atu1419, 10 μM Atu1419 in the presence of 100 μM MEF and 20 μM Atu1419-H3A mutant was performed by DSC on an auto PEAQ DSC (Malvern, France) in a standard buffer. Each measurement was preceded by a baseline scan with the standard buffer. Scans were performed at 1 K min^−1^ between 20 and 90°C. The heat capacity of the buffer was subtracted from that of the protein sample before analysis. Thermodynamic parameters were determined by fitting the data to the following equation:

$$\begin{equation*}\Delta {{\boldsymbol{C}}_{\boldsymbol{p}}}({\boldsymbol{T}}) = \frac{{{{\boldsymbol{K}}_{\boldsymbol{d}}}\left( {\boldsymbol{T}} \right){\boldsymbol{\ }}\Delta {{\boldsymbol{H}}_{{\boldsymbol{cal}}}}{\boldsymbol{\ }}\Delta {{\boldsymbol{H}}_{{\boldsymbol{vH}}}}}}{{{{\left[ {1 + {{\boldsymbol{K}}_{\boldsymbol{d}}}\left( {\boldsymbol{T}} \right)} \right]}^2}{\boldsymbol{\ R}}{{\boldsymbol{T}}^2}}}\end{equation*}$$

where *K*_d_ is the equilibrium constant for a two-state process, Δ*H*_vH_ is the enthalpy calculated on the basis of a two-state process and Δ*H*_cal_ is the measured enthalpy.

### Isothermal titration microcalorimetry measurements (ITC)

Isothermal titration microcalorimetry experiments were performed with an ITC200 isothermal titration calorimeter from MicroCal (Malvern, Orsay, France). The experiments were carried out at 20°C. Protein concentration in the microcalorimeter cell (0.2 ml) was 100 μM. Nineteen injections of 2 μl MEF solution at 1.2 mM were performed at intervals of 180 s while stirring at 500 rpm. The experimental data were fitted to theoretical titration curves with software supplied by MicroCal (ORIGIN®).

### Electron microscopy

Atu1419 (150 nM) was incubated with equimolar P*atu1418–1419* region (370 bp containing two palindromes separated by 190 bp) for 10 min at room temperature in a buffer containing 50 mM Tris pH 8 and 150 mM NaCl. Samples were deposited on a glow-discharged carbon coated grid and stained with 2% uranyl acetate. Images were recorded with a Lab6 Tecnai Spirit operating at 80 kVolt and a Quemesa Olympus CCD camera. Nominal magnification was X 45 000 corresponding to 3.17 Å/pixel.

## RESULTS AND DISCUSSION

### The transcriptional regulator Atu1419 is the second repressor of the HCAs degradation pathway

To study the role of Atu1419 in transcriptional regulation of the HCAs degradation genes, we constructed reporter fusions with pOT1e plasmids containing each promoter region of the SpG8-1b region (Figure 1) cloned upstream the *egfp* gene. This allowed reporting the transcription of each gene by measuring the eGFP fluorescence. The *atu1419* and *atu1420* plasmid reporter gene fusions were constructed in this study. Those corresponding to *atu1416* and *atu1418* were already available (11). The plasmid reporter fusions were introduced into the wild-type *A. fabrum* C58 strain to evaluate gene expression in response to ferulic acid. Compared to the fluorescence level measured in the absence of ferulic acid, the fluorescence level measured in the presence of ferulic acid was higher for the four reporter fusions (Table 2). Hence, *atu1416*, *atu1418*, *atu1419* and *atu1420* genes were induced in the presence of ferulic acid. The four plasmid reporter fusions were also introduced into the C58Δ*atu1419* mutant strain to compare genes expression with that of the wild-type C58 strain in the absence of ferulic acid. The fluorescence level measured after 24 hours for *atu1418*, *atu1419* and *atu1420* reporter fusions were respectively 3.67, 3.92 and 1.58 times higher in the C58Δ*atu1419* mutant strain than in the wild-type strain (Table 2). Thus, in the strain lacking Atu1419 regulatory protein (C58Δ*atu1419*), the reporter fusions for *atu1418*, *atu1419* and *atu1420* genes were constitutively expressed. In contrast, the fold change between C58Δ*atu1419* and the wild type strains was 0.63 and 0.97 for the *atu1416* gene fusion and the empty pOT1e vector, respectively indicating that *atu1416* gene expression was not repressed by Atu1419 (Table 2). These results showed that Atu1419 was a transcriptional repressor that regulates *atu1418*, *atu1420* and its own transcription.

**Table 2.** SpG8 1-b genes expression in the wild-type (WT) and C58Δ*atu1419* strains. Values refer to fold change at 24 h obtained by comparison of genes expression in the WT C58 strain with and without 750 μM of ferulic acid and correspond to the mean of three biological replicates with at least six technical replicates. Fold change at 24 h without ferulic acid was obtained by comparison of genes expression in the C58Δ*atu1419* and the WT strains and corresponds to the mean of at least two biological replicates and six technical replicates. Empty pOT1e corresponds to plasmid without any promoting region before the *egfp* gene in order to measure basal expression of the system

Genes	Fold change C58 WT in the presence/in the absence of ferulic acid	Fold change C58Δ*atu1419*/WT in the absence of ferulic acid
*Patu1416*	2.53 ± 0.2	0.63 ± 0.08
*Patu1418*	1.44 ± 0.19	3.67 ± 0.14
*Patu1419*	1.81 ± 0.13	3.92 ± 0.12
*Patu1420*	3.31 ± 0.32	1.58 ± 0.26
Empty pOT1e	1.12 ± 0.11	0.97 ± 0.05

### DNA-binding targets

**Figure 2.**
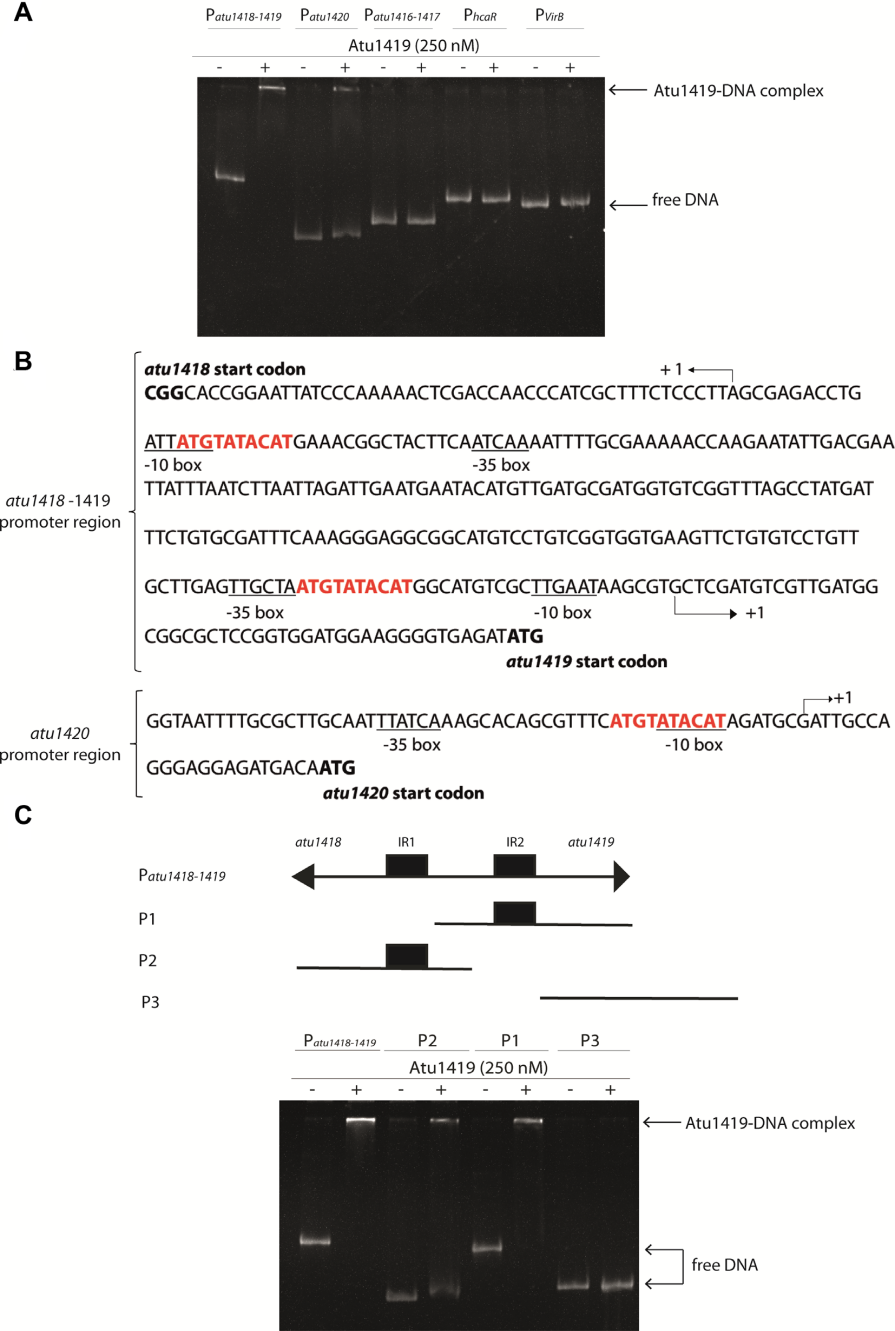
Atu1419 regulation mechanism. (**A**) EMSA analysis of 30 nM of each promoter region P*atu1416–1417*, P*atu1418–1419*, P*atu1420*, P*hcaR* (*atu1422*) incubated without and with 250 nM tetrameric Atu1419 (ratio protein:DNA of 8.3). P*virB* was used as a promoter region control. (**B**) In silico analysis of the *atu1418–1419* and *atu1420* promoter regions performed with the BPROM program (31): –10 and –35 boxes are underlined, and the palindromic sequences are shown in red. The transcription initiation sites are shown with an arrow and the translation start sites are indicated in bold. Palindromic regions (IR1 and IR2) are separated by 190 bp in *atu1418–1419* region. (**C**) EMSA analysis of 30 nM of three parts of the promoter region of *atu1418–1419* containing each one palindrome (P1 or P2) or none (P3) incubated without and with 250 nM tetrameric Atu1419 (ratio protein:DNA of 8.3).

To characterize the target DNA sequence of Atu1419, we purified Atu1419 (theoretical molecular weight of ∼26 542 Da for a monomer), which appeared as a tetramer in solution according to gel filtration/SEC-MALS chromatography (Supplementary Figure S1). Using the intergenic regions (between 300 and 400-base pair (bp)) of *atu1416–1417*, *atu1418–1419*, *atu1420*, *hcaR* and *virB* (negative control gene outside the SpG8-1b region) as DNA probes for binding assays (Figure 2A), we showed that Atu1419 bound to the *atu1418–1419* and *atu1420* promoter regions only, in agreement with the gene expression data described above.

In silico comparison of the promoter regions of *atu1418–1419* and *atu1420* using the BPROM program (31) revealed three 10-mer identical palindromic sequences (5′-ATGTATACAT-3′), two in *atu1418–1419* promoter region and one in the *atu1420* promoter region (Figure 2B). A palindromic sequence overlaps the -10 box regulatory element of *atu1418* and *atu1420*, whereas in the *atu1418–1419* promoter sequence, an additional palindromic sequence is found between the –10 and the –35 regulatory elements of the *atu1419* (Figure 2B). Using electrophoretic mobility shift assay (EMSA), we showed that Atu1419 was able to bind each palindrome of the *atu1418–1419* promoter sequence without the presence of the other palindrome meaning that only one palindrome site was required for binding (Figure 2C). Nonetheless, the intensity of the shifted bands was greater for the DNA fragment containing both palindromes (Figure 2C). We also analyzed the quaternary structure of Atu1419 in complex with the 10-bp palindrome using gel filtration/SEC-MALS measurements (Supplementary Figure S1). Atu1419 was also tetrameric upon DNA binding.

### Crystal structures of Atu1419 in complex with a fortuitous ligand

**Figure 3.**
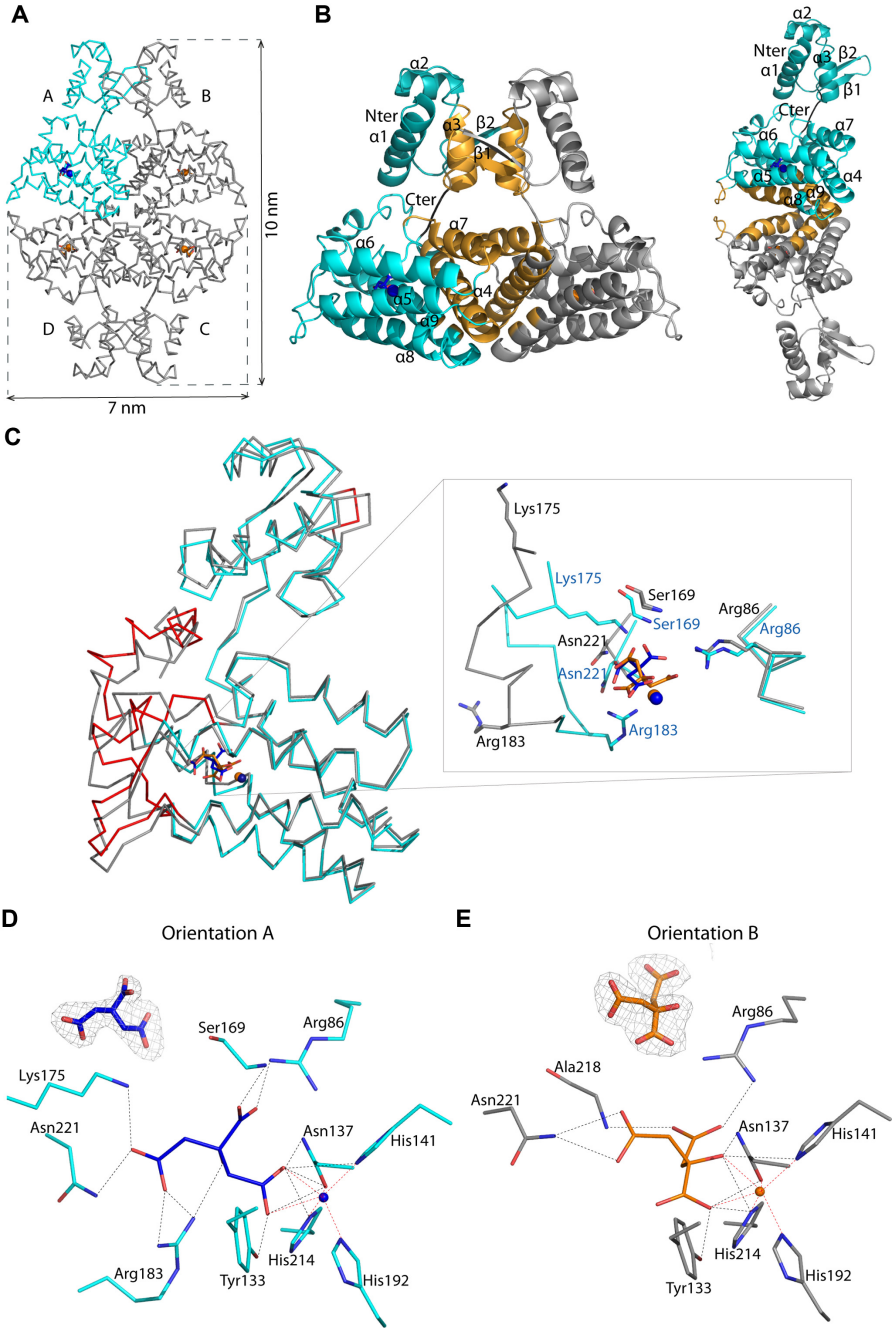
(**A**) Wire representation of Atu1419 tetramer in complex with a fortuitous citrate molecule and a co-purified Zn^2+^ ion, shown as sticks and ball, respectively, bound to the effector binding domain in the structure at 2 Å resolution (molecule A is in cyan whereas molecules B/C/D in gray). (**B**) Left: ribbon representation of dimer AB within the tetramer in (A). The secondary structural elements are indicated in subunit A and the orange elements form the dimeric interface. The linker joining the N-terminal DNA binding domain and the C-terminal effector binding domain is shown in black. Right: ribbon representation of dimer AD within the tetramer in (A). The secondary structural elements are indicated in subunit A and the orange elements form the other dimeric interface (**C**) Superposition of the subunits A in cyan and B in gray of the tetramer in (A) (the subunits B–D adopt the same fold). The red parts indicate major conformational changes between both subunits. A close-up view of the citrate and ion binding sites in the effector/metal binding domain shown in blue and orange for subunits A and B, respectively. (**D**) Interactions between the bound citrate in orientation A and in blue/Zn^2+^ (blue ball) and subunit A in cyan. Hydrogen bonds are shown as dashed lines in black (distance below 3.2 Å) and metal contacts are shown as dashed lines in red. Residues involved in the interactions are labeled and shown as sticks. Citrate is shown in its Fo-Fc omit map contoured at 4σ in subunit A. (**E**) Interactions between the bound citrate in orientation B and in orange/Zn^2+^ (orange ball) and subunit B in gray. Hydrogen bonds are shown as dashed lines in black (distance below 3.2 Å) and metal contacts are shown as dashed lines in red. Residues involved in the interactions are labeled and shown as sticks. Citrate is shown in its Fo-Fc omit map contoured at 4σ in subunit A.

We solved the first structure of Atu1419 from crystals grown in sodium citrate buffer using SAD method at the peak absorption energy of Zinc at 2 Å resolution in the space group *P2_1_2_1_2_1_* (Table 1). The zinc ion comes from *E. coli* protein expression because no metal was added during protein purification and crystallization. Four molecules (A, B, C and D) are present in the asymmetric unit and form a tetramer (a dimer of dimer) (Figure 3A), which is consistent with the observation that Atu1419 is a tetramer in solution (Supplementary Figure S1). Each monomer consists of an N-terminal DNA binding domain (residues 1–71) and a C-terminal all α-helical effector binding domain (residues 76–244) (Figure 3B). The DNA binding domain is composed of three helices (α1–α3) with helix α2 and helix α3 forming the helix-turn-helix motif and two anti-parallel β-strands (β1–β2) connected by a small loop designated as the wing motif. The secondary element with six helices (α4–α9) and topology of the C-terminal domain place Atu1419 in the VanR group of the FCD subfamily of GntR transcriptional regulators. Approximately 2776 Å^2^ of accessible surface area is buried upon dimerization for dimers AB or CD corresponding to an average of 11.7% of the total surface area of each monomer and 41 amino acids per monomer. The interface between dimers AB and CD involves side chains of both domains: helix α3 and strand β1 (residues 49–67) of the DNA binding domain and the three helices α4 (residues 78–100), α7 (residues 150–163) and α9 (residues 208 and 212) of the effector binding domain (Figure 3B). Arg72 in the linker region participates to the dimer interface. Two salt bridges with Glu57-Arg63′ (the prime refers to the second monomer in the dimer), and two polar interactions Glu57-Ser53′ and Asn62-Arg155′ are located in the N-terminal domain whereas fourteen H-bonds/salt-bridges including Asp81, Glu84, Arg95, Arg100, Ser150, Arg155, Glu156, Glu208 and Arg212 belong to the C-terminal domain. The contacts of dimer AD through eleven polar interactions/salt bridges bury 686 Å^2^ per subunit and concern 23 amino acids, which are located in the loop between helices α5–α6 (mainly residues 123–125), helices α8 (residues 183–194) and α9 (residues 203–216) (Figure 3B). The dimer BC displays a smaller interface of 557 Å^2^ per subunit comprising 21 amino acids and the same structural elements as for dimer AD. The total surface contact area between two neighbouring subunits within the tetramer is characteristic of biological interactions (32).

Remarkably, the four subunits within the tetramer are not identical: molecule A shows large conformational changes as indicated by an average root mean square deviation (RMSD) of 1.28 Å for all Cα atoms compared with the subunits B/C/D, which are more similar (average RMSD of 0.7 Å for all Cα atoms). This is due to the presence of a citrate originated from the crystallization condition, which binds molecule A in the effector binding site in a different orientation (orientation A; Figure 3C and D) than that for citrates in subunits B/C/D (orientation B; Figure 3C and E), which bind similarly (Figure 3C). Four loop regions (shown in red in Figure 3C), comprised of residues 64–67 of the wing between β1 and β2 of the wHTH motif, residues 119–130 between helices α5 and α6, residues 169–184 between helices α7 and α8 and residues 225–244 corresponding to the end of the effector binding domain, can move between 3 and 10 Å. The loop region 169–184 can drastically rearrange upon ligand binding. In molecule A, the Cα atoms of Ser169, Lys175 and Arg183 are respectively, 0.92, 6.84 and 7.34 Å away from those in molecules B/C/D, allowing the side chains of Lys175, Arg183 and the NH main chain of Ser169 to interact with the citrate molecule (Figure 3C and D). These latter protein-citrate interactions cannot exist in subunits B/C/D. Nonetheless, all bound citrates share interactions with Arg86, Tyr133, Asn137, His141, His214, Asn221 and a Zn^2+^ ion that co-purified with Atu1419 (Figure 3D and E). Thirteen polar interactions are observed between monomer A and the citrate molecule (orientation A), which is buried within the monomer, leaving only 26.6 Å^2^ or 8.4% of the molecule surface exposed to solvent. In contrast, there are only ten polar interactions between monomers B/C/D and the citrate with a similar buried surface area.

The orientation B of the citrate was also observed in another structure of Atu1419 solved at a higher resolution of 1.75 Å but in a different space group (*P2_1_2_1_2*). Here, two similar monomers (RMSD of 0.98 Å for all Cα atoms) are in the asymmetric unit and form a dimer, which in turn form a tetramer by the crystal symmetry with globally unchanged dimer interfaces (1553 and 701 Å^2^ per subunit for dimers AB and AD, respectively) compared with those from the *P2_1_2_1_2_1_* structure. They resemble subunits B/C/D of the *P2_1_2_1_2_1_* structure and bind similarly a citrate molecule. Nonetheless, their citrates are slightly shifted by 0.6 Å toward Arg86 losing the interaction with Asn221 compared with those in the *P2_1_2_1_2_1_* structure (Supplementary Figure S2).

### Crystal structures of Atu1419 in complex with the palindromic DNA

**Figure 4.**
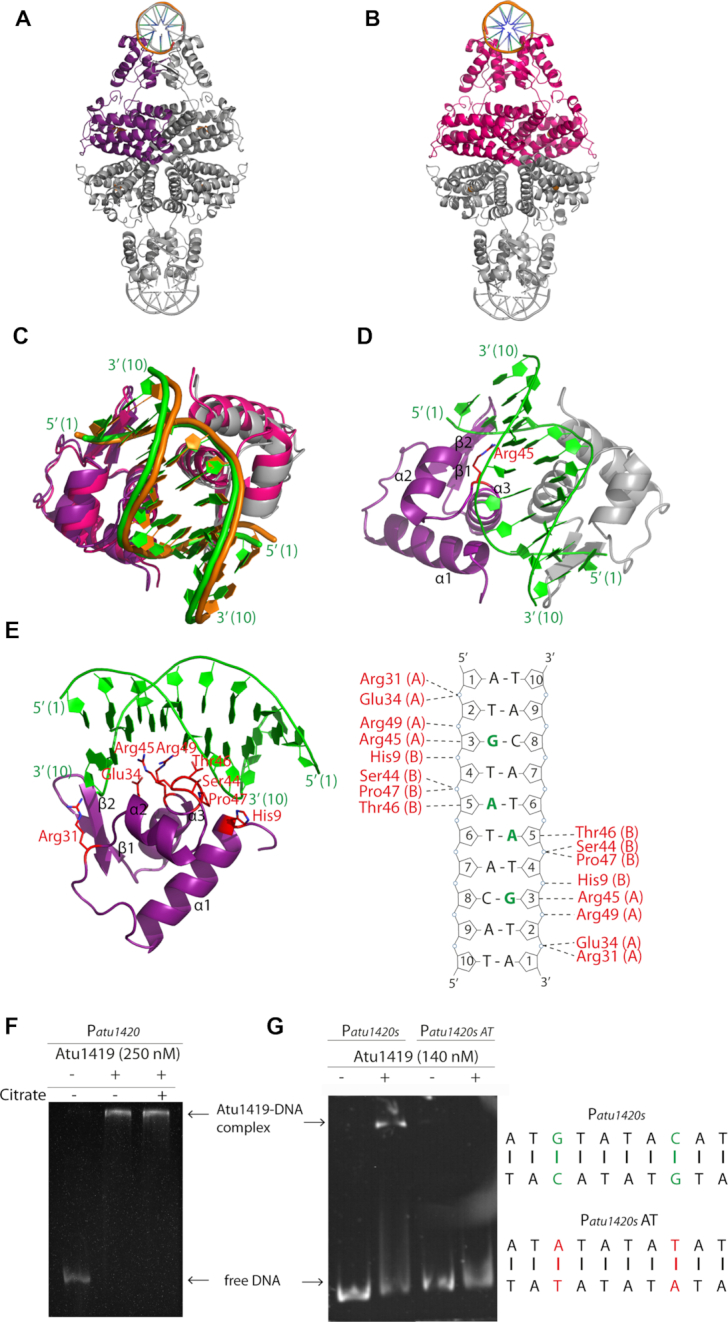
Cartoon representation of the tetrameric Atu1419-DNA complex (**A**) in the *P6_4_22* structure with the monomer of the asymmetric unit shown in purple. (**B**) in the *P2_1_2_1_2_1_* structure with the dimer of the asymmetric unit shown in magenta. (**C**) Superposition of the DNA binding domains of the dimers AB of the P6_4_22 structure in purple and gray for subunits A and B, respectively and of the *P2_1_2_1_2_1_* structure in magenta. The palindromic 10-mer DNA is in green and orange in the *P6_4_22* and *P2_1_2_1_2_1_* structures, respectively. (**D**) View of the DNA binding domain of the dimer AB of the *P6_4_22* structure in purple and gray for subunits A and B, respectively bound to the palindromic 10-mer DNA shown in green. The secondary elements are indicated in subunit A. Arg45 in helix α3 is the key residue, which interacts with the essential guanine base at position 3. This residue is shown as sticks. (**E**) View showing a close-up of the Atu1419–DNA interface within a monomer and schematic diagram of Atu1419–DNA contacts. Nucleotide bases that interact with Atu1419 are shown in green. (**F**) EMSA analysis of P*atu1420* incubated without and with Atu1419, and with Atu1419 and 100 μM citrate (ratio tetrameric protein:DNA of 8.3). (**G**) EMSA analysis of P*atu1420s* containing a unique palindrome or P*atu1420s* AT, in which the palindrome was mutated with the recognized guanine at position 3 replaced by an adenine and its cytosine partner replaced by a thymine, incubated without and with Atu1419 (ratio tetrameric protein:DNA of 4.6).

The structures of two palindromic DNA-Atu1419 complexes solved at 2.79 Å resolution (*P6_4_22* space group) and 2.05 Å resolution (*C222_1_* space group) present a distinct neighboring crystal packing with an asymmetric unit containing a monomer bound to a single DNA strand and a dimer bound to the 10-mer palindromic DNA, respectively (Table 1, Supplementary Figure S3). Nonetheless, a tetramer, where dimers AB and CD bind each to a DNA palindrome, was reconstituted by crystal symmetries (Figure 4A and B) in line with the conservation of Atu1419 tetramer in solution upon DNA binding. The three monomers of the asymmetric units (one in *P6_4_22* and two in *C222_1_*) are similar with an average RMSD value of 1 Å for all Cα atoms making almost identical interactions with DNA, although the positions of the DNA binding domains within the dimers between the two crystal structures do not completely overlap (Figure 4C). This observation explains the different crystal packing resulting of flexibility from both the DNA ligand and the DNA binding domains of Atu1419. Each monomer recognizes a half-site DNA with helices α2 and α3 of the HTH motif making nine polar interactions with both strands in the major groove of the DNA (Figure 4D and E). Helix α3 via Ser44, Thr46, Arg45 and Arg49 side chains is responsible for six hydrogen bonds with both strands, half with oxygens of phosphate groups 5′ of guanine (G3) at position 3 on one strand and of adenine (A5) at position 5 on the complementary strand. The other half is made by the guanidinium group of Arg45, which provides specific contacts with the N7 and O6 atoms of G3 on one strand and by OG1 of Thr46 with the N7 of A5 on the other strand (Figure 4C). The remaining H-bonds come from helix α2 *via* the Arg31 side chain and the main chain amino group of Glu34, and an additional interaction is present with the main chain NH of His9 in helix α1. They consist of phosphate contacts with A1 and T2 on one DNA strand and T4 on the other.

All Atu1419-DNA subunits contain a bound Zn^2+^ ion at the same position as that observed in both Atu1419–citrate complexes. In contrast to the protein-DNA complex in *C222_1_* space group, which crystallized in MES buffer, the *P6_4_22* protein–DNA complex reveals a bound citrate in the effector binding pocket. This citrate adopts the orientation B described in both Atu1419-citrate complexes, except for molecule A (orientation A) in the *P2_1_2_1_2_1_* structure. Because both tetrameric Atu1419-DNA structures are similar, the presence of the bound citrate in orientation B in all subunits of one DNA complex has clearly no effect on Atu1419 for DNA binding and DNA release, in agreement with EMSA (Figure 4G) and promoter activity (Supplementary Table S3).

We modified the specifically recognized guanine base G3 by an adenine base and its partner base C8 by a thymine on each strand within the unique palindrome of the *atu1420* promoter region. EMSA showed that Atu1419 was no longer able to bind P*atu1420s* AT mutant (Figure 4F). Therefore, the guanine at position 3 has an essential role in the DNA palindrome binding by Atu1419 meaning that Arg45 has a key role in recognizing this guanine base.

### Crystal structure of apo Atu1419 and structural comparison

**Figure 5.**
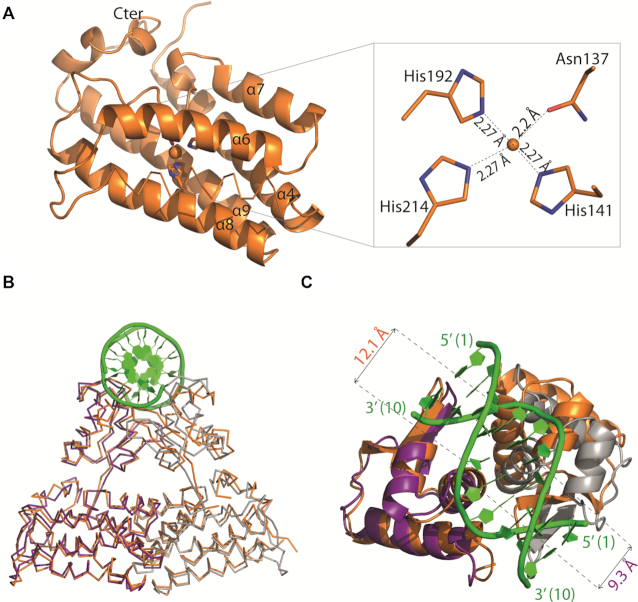
(**A**) Cartoon representation of the C-terminal domain except for helix α5 shown in ribbon of Atu1419 with a co-purified Zn^2+^ ion and a close-up view of the Zn^2+^ binding site. The four amino acid residues bound to the metal ion (orange ball) are shown as sticks. (**B**) Superposition of dimers AB of apo Atu1419 (in orange) and Atu1419-DNA complex (in purple and gray). The DNA is in green. (**C**) Close-up view showing the superposition of their DNA binding domains colored as in (B) with a view at 90°.

We solved the structure of Atu1419 in the apoform at 2.7 Å resolution using Tris-HCl buffer instead of citrate buffer in the crystallization condition to avoid any bound citrate (Table 1). The asymmetric unit contains a tetramer with a bound Zn^2+^ ion in each effector binding domain. Asn137 and three histidine (His141, His192 and His214) side chains form the metal binding site of Atu1419 and chelate the zinc ion with a classical tetrahedral coordination geometry and average distances ∼2.2 Å (Figure 5A). We mutated the three histidine (His141, His192 and His214) into alanine to make the triple point mutant Atu1419-H3A and alter the metal binding site. The zinc ion holds helices α6, α8 and α9 involved in the tetrameric interface far from the DNA binding domain, and likely interacts with the effector as shown by the structures solved with a citrate taking the place of the effector. Atu1419-H3A is correctly folded as checked by using circular dichroism (Supplementary Figure S4) and shares the same secondary structure content (about 58% α-helices and 1% β-sheets) as the wild-type protein in line with what is observed in all crystal structures of Atu1419. Similar to the wild-type protein, this mutant is tetrameric and capable of binding *atu1418–1419* and *atu1420* promoter regions.

The dimeric (dimers AB/CD) and tetrameric interfaces (dimers AD/BC) covering around 1400 Å^2^ and 516 Å^2^ per subunit, respectively, are comparable to those of Atu1419-citrate complexes and Atu1419–DNA complexes. Subunits A and D overlap well (RMSD of 0.53 Å), as well as subunits B and C (RMSD of 0.83 Å), whereas subunits A and B or C and D show a RMSD over 2 Å, mainly due to differences in the position of their HTH motif and the end of their C-terminal domain, evidencing large flexibility (Supplementary Figure S5A). This is confirmed by the structural comparison of the DNA binding domains of the apo dimer AB with those of the Atu1419–DNA complex (Figure 5B). While subunits A are rather similar, helix α3 of the apoform subunit B clashes into the DNA sugar-phosphate backbone. Indeed, this helix is far from its optimized position for DNA binding, which is perpendicular to the helical axis of the DNA. A displacement up to 7 Å would be required to correctly places its wHTH motif. A similar structural analysis between the DNA binding domains of the Atu1419-citrate complexes and Atu1419–DNA complexes led to similar conclusions (Supplementary Figures S5B–D).

It is noteworthy that the conformation of the effector binding pocket in all subunits bound or not to DNA and containing a citrate in orientation B is similar to that of the empty pocket observed in the apo form and one DNA complex. This suggests that this citrate may mimic a non-productive-like binding in the effector binding site.

### Comparison with other FCD members

Atu1419 belongs to the VanR subgroup of the FCD subfamily of the large GntR superfamily due to the number of helices in its effector binding domain. The FCD subfamily is divided into two groups; VanR and FadR. Members that are structurally characterized in FadR subgroup possess an additional helix after the linker region at the beginning of the effector binding domain (23,33–35). This major difference leads to a domain-swapped quaternary structure, in which the DNA binding domain of FadR subunit A crosses the dimerization interface to be in contact with the effector binding domain of subunit B (35–37). This is not observed in the VanR subgroup where both domains from the same subunit are associated.

Atu1419 is now the seventh VanR member to be structurally characterized. The six others are *Thermotoga maritima* TM0439 (PDBs 3SXY/3FMS (38)), *Ralstonia eutropha* JMP134 YP_298823.1 (PDB 3IHU no related publication), *Pseudomonas syringae* PS5454 (PDB 3C7J, no related publication), *Rhodococus* sp. *RHA1* (PDB 2HS5, no related publication), *E. coli* McbR/YncC (PDB 4P9F (37)) and *Listeria monocytogenes* MouR (PDB 6EP3 (39)). Structural comparison using SSM-EBI (http://www.ebi.ac.uk/msd-srv/ssm) reports RMSD of 3.19 Å over 190 Cα atoms/3.46 Å over 184 Cα atoms between the full-length of Atu1419 and MouR (PDB 6EP3)/TM0439 (PDB 3SXY), respectively. The same search using the C-terminal domain of Atu1419 only improves the RMSD values to 2.59 and 2.71 Å over 127 residues with the effector binding domains of TM0439 and MouR corresponding to 15% and 13% sequence identity, respectively (Supplementary Figure S6A). The best match concerns the effector binding domain of McbR/YncC (PDB 4P9F) with a RMSD of 2.23 Å over 132 residues and 18% sequence identity, meaning that there are major significant differences between Atu1419 and the six other VanR members. While only the C-terminal domain is responsible for dimerization for most VanR regulators (37), Atu1419 is similar to McbR/YncC in using both the N- and C-terminal domains as the dimeric interface. However, the dimer of Atu1419 does not structurally resemble that of McbR/YncC (Supplementary Figure S6B).

Like Atu1419, two other VanR members, which are TM0439 and PS5454 display a metal binding site with three histidine residues and an asparagine or an aspartate residue involved in the metal coordination (37,38). These histidine residues are conserved and structurally close (Supplementary Figure S6C). LldR (PDB 2DI3) from the FadR group binds a zinc ion chelated by these three conserved histidines and an aspartate (35). It was proposed that an additional subgroup within the FCD subfamily could be created for regulators from both FadR and VanR members, which are capable of binding to metal ions (35,38). Using thermal denaturation experiments by differential scanning calorimetry (DSC), *T*_m_ of 52.6 and 34°C were measured with the wild-type Atu1419 and Atu1419-H3A mutant respectively (Supplementary Figure S7). The drastic difference of almost 20°C indicates that the metal assists in stabilizing the structure affording an explanation as to why Atu1419-H3A could not be concentrated over 50 μM without precipitating, in contrast to the wild-type protein (140 μM). All attempts to crystallize Atu1419-H3A were unsuccessful.

Atu1419 is the first VanR member to be structurally characterized in complex with DNA whereas several FadR-DNA complexes are available (23,33,34). Each dimer of Atu1419 within the tetramer can bind an identical palindrome. Similarly to regulators with a wHTH motif such as MarR regulators (40,41), FCD regulators bind to one half-site of the palindromic DNA, with the dimerization interface helping to establish the spacing between the two half-sites. The DNA binding domains of Atu1419, which are involved in the dimerization interface, allow Atu1419 to bind a short palindrome of 10 base pairs. As in *E. coli* FadR (PDB 1H9T (23) and 1HW2 (33)) and in *Vibrio cholerae* FadR (PDB 4P9U (34)), the DNA-binding site of each Atu1419 monomer is recognized by conserved residues such as Glu34 in helix α2, Arg45 (specific DNA contacts), Thr46 and Arg49 (phosphate backbone contacts) in helix α3 that interact with the major groove and Gly66 in the tip of the wing (residues 64–68) between β1 and β2. Nonetheless, the wing in Atu1419 does not appear as crucial for DNA interaction as that in FadR, which recognizes a pseudo-palindrome of 17 base pairs. Indeed, His65, which specifically recognizes a DNA base in FadR is a glycine in Atu1419. DNA binding domains superposition of the Atu1419-DNA complex with those of *E. coli* and *V*. *cholerae* FadR-DNA complexes shows that although the positions of subunits A are rather similar, the second subunit of Atu1419 makes steric clashes with the DNA bound to FadR and that of FadR is too far from the DNA bound to Atu1419 (Supplementary Figure S8). Atu1419 displays only one specific interaction shared in FadR with the guanine present within the major groove of DNA *via* their conserved Arg45, a typical feature of the GntR family (22). The other specific DNA contacts of FadR (via Arg35, Thr44, Thr46 and His65) are not comparable with those of Atu1419. The recognized DNA sequence of Atu1419 which is 5′-ATGTATACAT-3′ is in agreement with the predicted DNA signature for the GntR family: 5′-(N)_*y*_GT(N)_*x*_AC(N)_y_-3′ where the number *x* and *y* vary (20). Later, this DNA binding signature was suggested to be modified as 5′-TNG(N)nCNA-3′ on the basis of base-specific interactions (22) or as 5′-TGGTNxACCA-3′ for FadR subgroup (35) but both sequences are not appropriate for Atu1419.

The DNA binding of Atu1419 requires conformational changes of the DNA binding domains. Indeed, the distance between the two DNA recognition helices (Thr46 of helix α3) narrows from 12 Å in the apoform to 9.3 Å in the Atu1419-DNA complex, for a productive interaction with DNA (Figure 5C). A similar observation was reported for *V. cholerae* FadR (PDB 4P9U (34)). The structures of FadR repressors in complex with their effector show significant conformational changes transmitted from the effector binding domain to the DNA binding domain, leading to a conformational state that is no longer favorable for interaction with DNA (22,23,34). The fundamental process wherein the binding of a ligand or effector molecule alters the activity of the protein at a distant site is defined as an allosteric mechanism. In the case of Atu1419, the citrate in orientation A bound in the effector binding pocket induces large conformational changes from the C-terminal domain to the N-terminal domain. It affects four different protein regions: the wing of the DNA binding domain and three regions of the effector binding domain including the C-terminus protein which is in contact with the DNA binding domain (Figure 3C). The presence of this citrate clearly reveals the plasticity of the effector binding pocket and the large potential rearrangement of the repressor. It also shows that within the tetramer, one subunit can adopt a different conformation around the effector binding pocket and further away (Figure 3C) compared to the three others subunits, highlighting an allosteric mechanism. The citrate bound in orientation A may mimic a part of the physiological effector, which binds to the metal ion.

### Atu1419 effector is the N5,N10-methylenetetrahydrofolate (MEF)

The effector molecule bound to the members of the GntR superfamily are often related to catabolic substrates or intermediates of the pathway controlled by the transcription factor (19,20), and several molecules are produced during the ferulic acid degradation pathways (5). Because the citrate molecule was able to bind to the effector binding site of Atu1419, we searched for molecules resembling citrate among those described in the HCA pathway (Figure 1A), and we asked whether the substrate-cofactor H4F or the product-cofactor M4HF of the O-demethylase Atu1420 enzyme could be a potential effector. Indeed, citrate compound with three carboxylate groups mimics two carboxylate groups located at one end of the H4F or MH4F molecules. Alternatively, MEF, the putative product of Atu1418 enzyme, which possesses the same two carboxylate groups could also be a potential effector of Atu1419.

**Figure 6.**
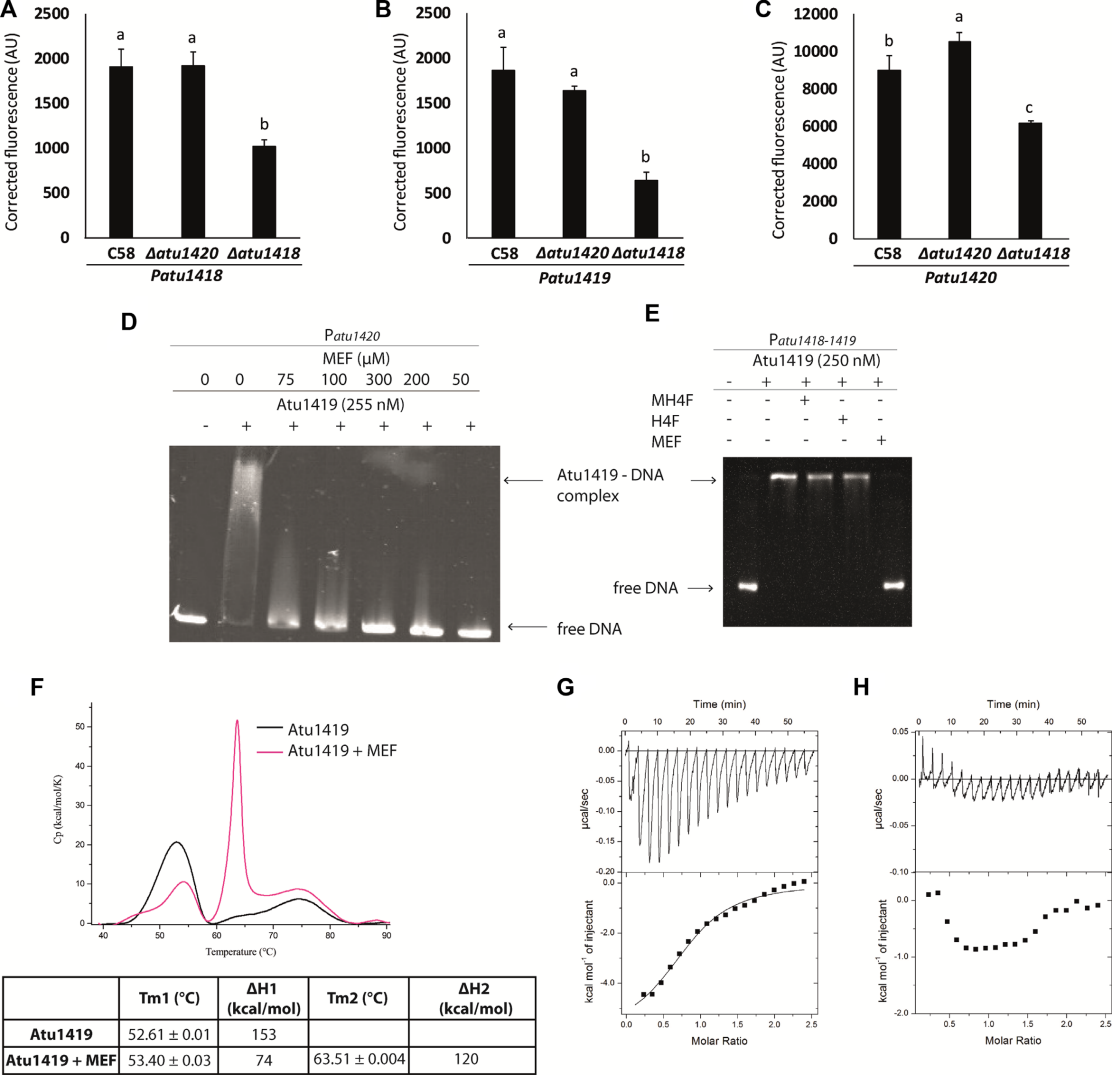
(**A**–**C**) P*atu1418*, P*atu1419* or P*atu1420* expression in *A. fabrum* C58 wild-type strain compared with that in C58Δ*atu1420* and C58Δ*atu1418* mutants in the presence of ferulic acid. Values correspond to normalized fluorescence intensity in the presence of ferulic acid corrected by subtracting values without ferulic acid. Different letters indicate statistical differences between conditions (one-way ANOVA and Tukey test; *P*-value < 0.05). (**D**) EMSA analysis of P*atu1420* DNA region incubated without and with Atu1419 (ratio tetrameric protein:DNA of 17) and N5,N10-methylenetetrahydrofolate at different concentrations (0–300 μM). (**E**) Gel mobility shift assay analysis of P*atu1420* DNA region incubated without and with Atu1419, and with Atu1419 and H4F, MH4F or MEF at 100 μM. (**F**) Differential scanning calorimetry thermograms of apo Atu1419 (black) and Atu1419 in the presence of MEF (magenta). The table below indicates the T*m*. DSC experiments were performed twice. (**G**) Isothermal titration microcalorimetry (ITC) experiments of Atu1419 towards MEF. The top panel shows heat differences upon injection of MEF and low panel shows integrated heats of injection with the best fit (solid line) to a single binding model using Microcal ORIGIN. (**H**) Control of MEF injection in the buffer solution by ITC.

To verify this, P*atu1418, Patu1419* and P*atu1420* plasmid reporter gene fusions were introduced into the C58Δ*atu1418* and C58Δ*atu1420* mutant strains. For each strain, the fluorescence level was monitored in the presence or absence of ferulic acid and compared to the wild-type *A. fabrum* C58 (Figures 6A–C). For the C58Δ*atu1418* strain, the overproduction of fluorescence observed in the presence of ferulic acid is significantly lower than that in the wild-type C58 and C58Δ*atu1420* strains. These results indicate that the presence of *atu1418* gene is important for the full induction of *atu1418*, *atu1419* and *atu1420* genes expression in the presence of ferulic acid. The accumulation of MH4F and the lack of MEF production in the defective C58Δ*atu1418* mutant suggests that MEF could be the effector of Atu1419. MEF is not stable in aqueous *in vitro* solutions as it enters a rapid reversible equilibrium into H4F (42,43). Nonetheless, immediately using freshly-made MEF solution for EMSA, we were able to show that MEF could relieve repression of Atu1419 (Figure 6D) whereas H4F and MH4F had no effect (Figure 6E). In the C58Δ*atu1418* mutant, induction *of* P*atu1418, Patu1419* and P*atu1420* was not totally abolished, probably due to functional redundancy of N5,N10-methylene tetrahydrofolate reductase activity in *A. fabrum* genome, which allows to keep a basal pool of folate and intermediates essential for the cell (44).

The interaction between MEF and Atu1419 was confirmed by DSC with a *T*_m_ of 63.5°C for Atu1419 in complex with MEF compared to the *T*m of 52.6°C for the apo Atu1419 (Figure 6F). This was also confirmed by isothermal titration microcalorimetry (ITC, Figure 6G). Because MEF instability did not allow a return to the baseline between two injections, we could not rigorously determine a dissociation constant *K*_D_ value, which is around 20 μM. Injection of MEF towards buffer solution was a control of its instability effect (Figure 6H). No interaction could be observed between MH4F and Atu1419 by DSC and ITC, in agreement with the results from EMSA.

### Atu1419 tetramer is the biologically active form

We analyzed the quaternary structure of Atu1419 in complex with MEF using gel filtration measurement (Supplementary Figure S9). Atu1419 remains tetrameric when bound to MEF. Thus, Atu1419 is a tetramer in its apo form, upon DNA binding or effector binding suggesting that this quaternary structure is the biologically active form. Both crystal structures of Atu1419 in complex with DNA revealed that each dimer of Atu1419 within the tetramer can bind a palindromic site of 10 bp, separated by approximately 10 nm (Figure 3A). This separation distance is compatible with DNA loop formation (45) allowing the simultaneous binding of two distant palindromes (190 bp apart) within the intergenic region of *atu1418–1419*. Thus, Atu1419 tetramer could repress gene expression *via* DNA looping, such as several negative regulators in prokaryotes (46) such as the extensively studied tetrameric *lac* operon repressor (47).

**Figure 7.**
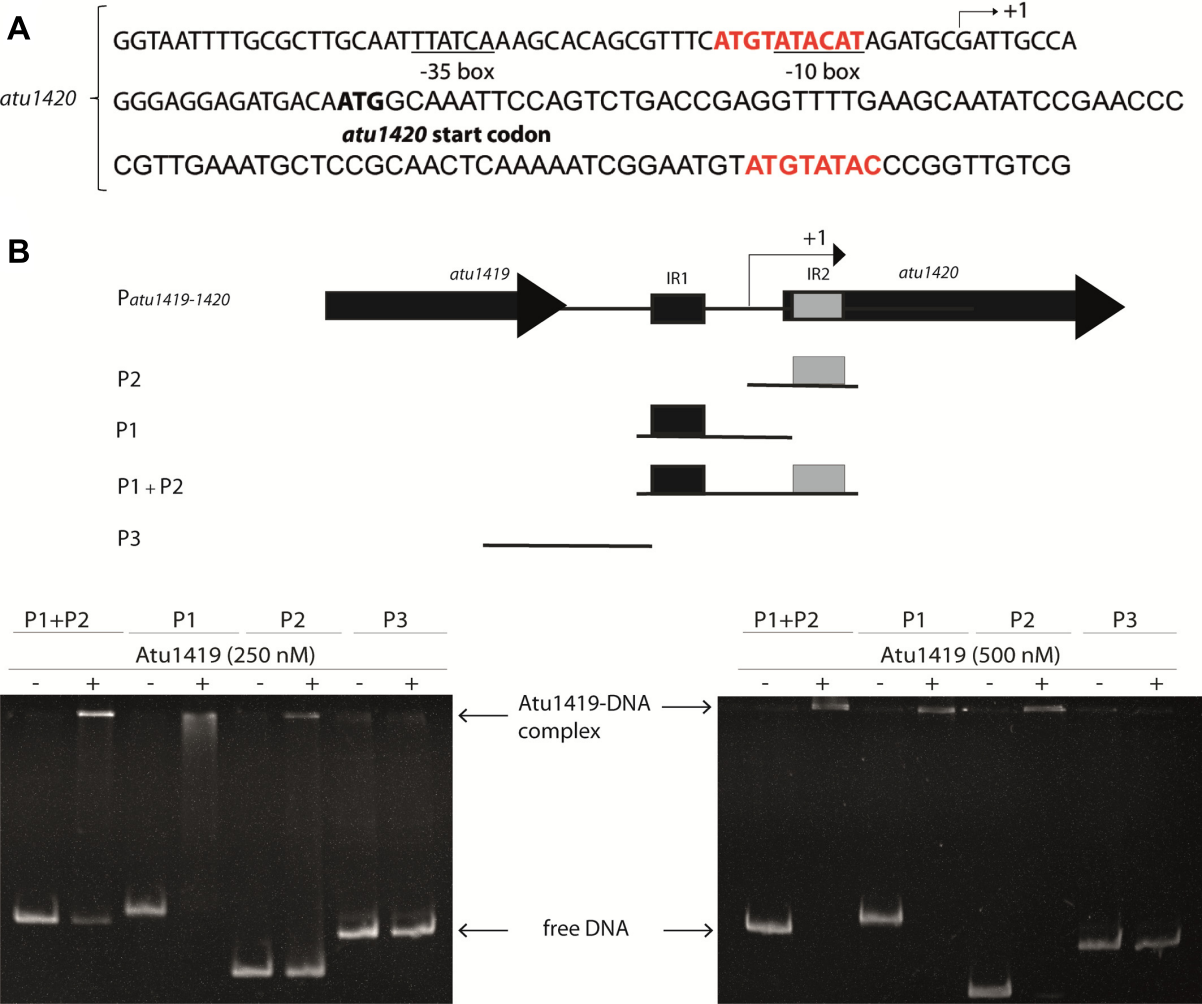
(**A**) *atu1420* promoter region: –10 and –35 boxes are underlined, the palindromic sequence and the degenerated palindromic sequence (109 bp apart) are in red. The transcription initiation site is shown with an arrow and the translation start site is indicated in bold. (**B**) Gel mobility shift assay analysis of four parts of the promoter region of *atu1420* containing each one palindrome or one degenerated palindrome (P1 or P2), or both (P1+P2) or none (P3) incubated without and with 250 or 500 nM tetrameric Atu1419 (ratio protein:DNA of 8.3 or 16.6).

We searched for a second potential DNA binding site of Atu1419 in *atu1420* region and found a degenerated palindrome (8 bp conserved over the 10 bp palindrome; Figure 7A). The degenerated palindrome is separated by 109 bp from the palindrome and is located downstream within the open reading frame of *atu1420* gene (Figure 7A). We then performed EMSA with three oligonucleotides containing either the palindrome, the degenerated palindrome, or both and identified in the gel a retarded band for each oligonucleotide proving the formation of a stable non-covalent protein-DNA complex (Figure 7B). Thus, the repressor can recognize a palindrome and a degenerated palindrome providing it the opportunity to bind simultaneously to two DNA sites in each region (*atu1418–1419* and *atu1420*), in agreement with its oligomeric state. Similarly, the structure (a tetramer composed of dimer of dimer) of *Cupriavidus necator* CbnR transcriptional regulator from LysR family (PDBs 1IXC and 1IZL (48)) is compatible with an interaction with two DNA binding sites on a bended DNA fragment, where the authors suggested that the quaternary structure of CbnR should be changed to relax the bent DNA that binds to the CbnR tetramer (48). Conformational changes of one subunit of CbnR and Atu1419 can be easily propagated to the other parts of the tetramer through inter-subunit interactions. In contrast, the quaternary organization of Lac repressor (a tetramer comprising a pair of dimers loosely associated with each other *via* their C-terminal helices forming a four-helix bundle) suggests that the structural change of each dimer seems to be able to occur independently. Noteworthy, single molecule experiments revealed that a repressor securing a DNA loop is a stronger transcriptional roadblock for RNA polymerases than one bound to a single DNA site (49).

**Figure 8.**
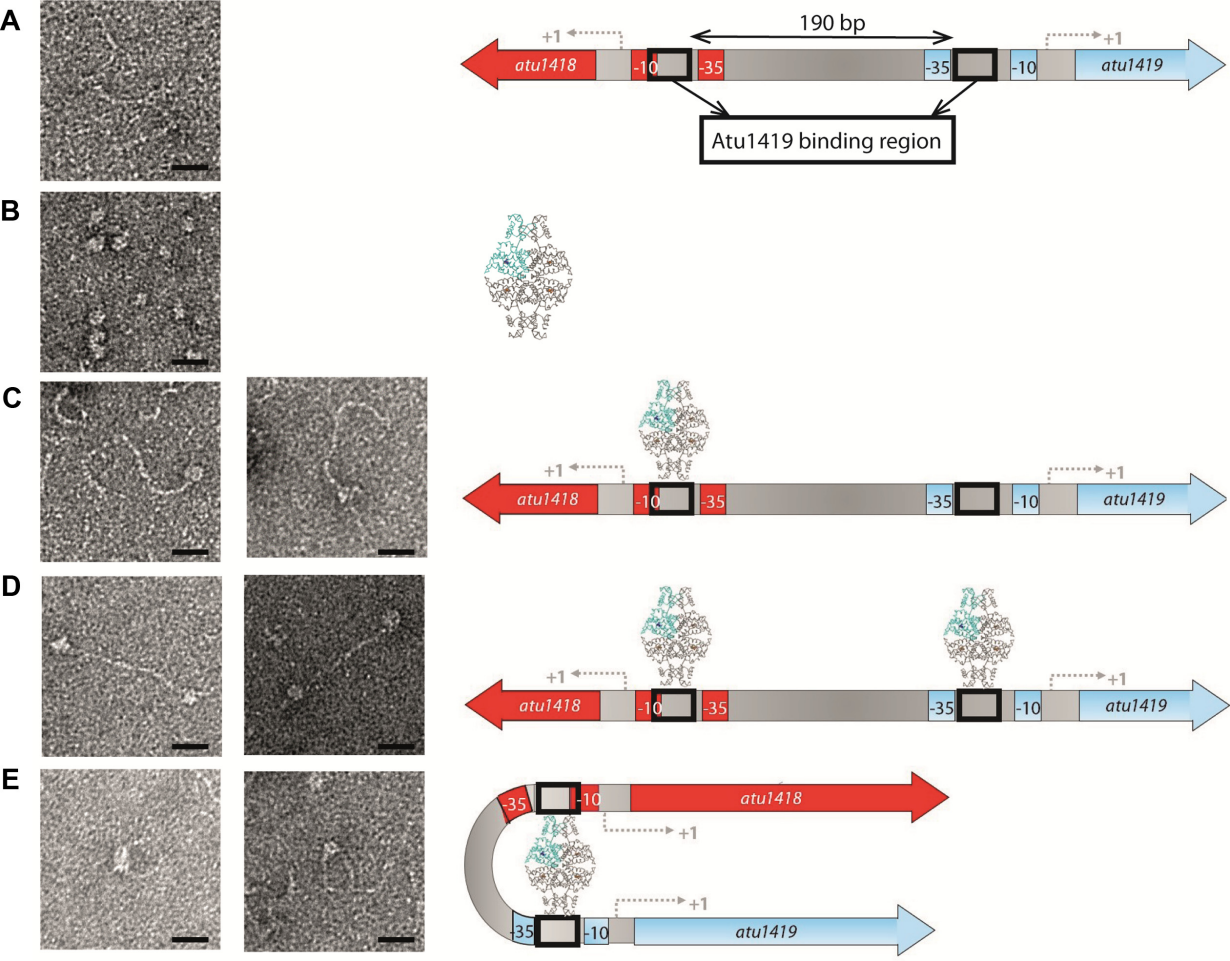
Interactions between Atu1419 repressor and P*atu1418–1419* region of 370 bp containing two palindromes separated by 190 bp visualized by electron microscopy on the left, and corresponding models on the right. (**A**) Image of P*atu1418–1419* region, (**B**) image of Atu1419 tetramer, (**C**) image of one tetramer bound to one palindrome, (**D**) image of two tetramers bound each to one palindrome, (**E**) image of one tetramer inducing a DNA loop. Scales bars: 20 nm.

Finally, we investigated the binding between Atu1419 and the P*atu1418–1419* region of 370 bp containing two palindromes (190 bp apart) using negative staining electron microscopy. We measured the length of the DNA fragment (∼125 nm) and the size of the protein (∼10 nm corresponding to a tetramer), which are in good agreement with the expected values (129 nm and 10 nm, respectively, Figure 8A and B). We were able to observe three categories of binding: a tetramer bound to one DNA fragment, two tetramers bound to one DNA fragment and a tetramer forming a DNA loop (Figure 8C–E). The loops were measured at ∼60–65 nm compatible with the length of 190 bp, which separates two palindromes.

## CONCLUSION

Hydroxycinnamic acids are involved in the initiation of the plasmid-encoded pathogenicity program of *A. fabrum* and can be degraded through the expression of the species-specific chromosomal gene cluster composed of eight genes, which are regulated by two repressors HcaR/Atu1422 and Atu1419. We have previously shown that HcaR, a dimeric protein from the MarR family, is the transcriptional repressor of its own transcription, *atu1421* gene and that of the first three genes (*atu1415*, *atu1416* and *atu1417*) of HCA degradation pathway. Here, we characterized Atu1419 as a transcriptional repressor of its own repression in addition to two other genes (*atu1418* and *atu1420*). We identified the short palindromic region composed of 10 base pairs and a degenerated palindrome bound to Atu1419 and the N5,N10-methylenetetrahydrofolate (MEF) as the effector molecule disrupting Atu1419-DNA interaction, allowing the expression of the second part of the HCA degradation pathway. Remarkably, Atu1419 repressor is not regulated by the direct catabolic substrates/intermediates of the pathway. An unexpected outcome was that Atu1419 is a tetrameric regulator whereas all GntR regulators so far studied have been shown to be dimeric (20–22). This quaternary structure allows the repressor to bind two distant DNA sites simultaneously making DNA loop repression (Figure 8) and providing a fine-tune mechanism in the transcriptional regulation of the appropriate genes at the right time. Our structural and biophysical study of Atu1419 revealed different structural rearrangements around the fortuitous citrate bound in the effector binding domain within the tetramer and a double role of the Zn^2+^ ion in protein stability and effector binding. This suggests an induced-allosteric mechanism by MEF, involving conformational changes ‘in cascade’ which enhance interactions between the DNA binding domain and the end of the effector binding domain to constrain the flexibility of the HTH motif to prevent it from DNA binding.

## DATA AVAILABILITY

The atomic coordinates and structure factors have been deposited at the Protein Data bank under PDB ID 6Z74 and 6ZA0 for Atu1419 in complex with a fortuitous citrate in *P2_1_2_1_2_1_* and *P2_1_2_1_2*, respectively, PDB ID 6ZAB and 6ZA3 for Atu1419 in complex with DNA in *P6_4_22* and *C222_1_*, respectively and PDB ID 6ZA7 for apo Atu1419.

## Supplementary Material

gkaa1181_Supplemental_File

## Notes

Present address: Anaïs Naretto, Department of Biochemistry, Vanderbilt University, Nashville, TN 37235, USA.
