# Supplementary material for: Characterization of the first tetrameric transcription factor of the GntR superfamily with allosteric regulation from the bacterial pathogen Agrobacterium fabrum
Source: Nucleic Acids Res. 2020 Dec 11;49(1):529–46. doi: 10.1093/nar/gkaa1181 (PMC7797058; doi:10.1093/nar/gkaa1181)
Supplement: gkaa1181_Supplemental_File [file gkaa1181_supplemental_file.pdf]

Supplementary material

Characterization of the first tetrameric transcription factor of the GntR superfamily with allosteric regulation from the bacterial pathogen *Agrobacterium fabrum*

Armelle Vigouroux[1]†, Thibault Meyer[2]†, Anaïs Naretto[1#], Pierre Legrand[3], Magali Aumont-Nicaise[1], Aurélie Di Cicco[4], Sebastien Renoud[2], Jeanne Doré[2], Daniel Lévy[4], Ludovic Vial[2], Céline Lavire[2*], Solange Moréra[1*]

1 Université Paris-Saclay, CEA, CNRS, Institute for Integrative Biology of the Cell (I2BC), 91198, Gif-sur-Yvette, France

2 Université Claude Bernard Lyon 1CNRS, INRAE, VetAgro Sup, UMR Ecologie Microbienne, F 69622 Villeurbanne, France

3 Synchrotron SOLEIL, L'Orme des Merisiers, Saint-Aubin, 91192, Gif-sur-Yvette, France

4 Laboratoire Physico Chimie Curie, Institut Curie, PSL Research University, CNRS UMR 168, Paris 75005, France

[#]Present Address: Anaïs Naretto, Department of Biochemistry, Vanderbilt University, Nashville, Tennessee, 37235, USA

† These authors contributed equally to this work

* To whom correspondence should be addressed. Tel: +33169824213; E-mail: solange.morera@i2bc.paris-saclay.fr; Tel: +33426237126; E-mail: celine.lavire@univ-lyon1.fr

Supplementary Material, Supplementary Tables S1-S3 and Figures S1-S9.

Table S1. Strains and plasmids used in this study

	Relevant genotype and description	Reference or source
Escherichia coli		
JM109	*endA1 glnV44 thi-1 relA1 gyrA96 recA1 mcrB+ Δ(lac-proAB) e14- [F' traD36 proAB+ lacIq lacZΔM15] hsdR17(rK-mK+)*	NEB catalog
BL21	$F^{–}$ *ompT gal dcm lon* $hsdS_B(r_B^{–}m_B^{–})$ $[malB^{+}]_{K\text{-}12}(\lambda^{S})$	NEB catalog
Stellar	*F–, endA1, supE44, thi-1, recA1, relA1, gyrA96, phoA, Φ80d lacZΔ M15, Δ (lacZYA - argF) U169, Δ (mrr - hsdRMS - mcrBC), ΔmcrA, λ–*	Takara Clontech
Agrobacterium fabrum		
C58	Wild-type	CFBP 1903
C58Δ*atu1418*	C58 deleted of *atu1418* gene	(1)
C58Δ*atu1419*	C58 deleted of *atu1419* gene	This study
C58Δ*atu1420*	C58 deleted of *atu1420* gene	This study
**Plasmids**		
pJQ200SK	Suicide vector; P15A sacB; Gm^{R}	(2)
pOT1e	Promoter-probe vector based on pBBR1MCS-5 replicon; contains promoterless eGFP and MCS between two transcriptional terminators; Gm^{R}	(3)
pET-20b	*E. coli* expression vector (6His-tag), Amp^{R}	Novagen
P*atu1416*-eGFP	Upstream region *atu1416* inserted in site ClaI-SalI in pOT1e; Gm^{R}	(4)
P*atu1418*-eGFP	Upstream region *atu1418* inserted in site ClaI-SalI in pOT1e; Gm^{R}	(4)
P*atu1419*-eGFP	Upstream region *atu1419* inserted in site ClaI-SalI in pOT1e; Gm^{R}	This study
P*atu1420*-eGFP	Upstream region *atu1420* inserted in site HindIII-PstI in pOT1e; Gm^{R}	This study
pET-20b-*atu1419*	*atu1419* inserted in site NdeI-XhoI in pET-20b, His-tag fusion, Amp^{R}	This study
pET-20b-*atu1419*-H3A	*atu1419* mutant (H92A, H141A and H214A) inserted in site NdeI-XhoI in pET-20b, His-tag fusion	This study
pJQ200SK-Δ*atu1419*	Suicide vector used for *atu1419* deletion; Gm^{R}	This study
pJQ200SK-Δ*atu1420*	Suicide vector used for *atu1420* deletion; Gm^{R}	This study

1. Campillo, T., Renoud, S., Kerzaon, I., Vial, L., Baude, J., Gaillard, V., Bellvert, F., Chamignon, C., Comte, G., Nesme, X. et al. (2014) Analysis of hydroxycinnamic acid degradation in *Agrobacterium fabrum* reveals a coenzyme A-dependent, beta-oxidative deacetylation pathway. Appl Environ Microbiol, 80, 3341-3349.
2. Quandt, J. and Hynes, M.F. (1993) Versatile suicide vectors which allow direct selection for gene replacement in gram-negative bacteria. Gene, 127, 15-21.
3. Allaway, D., Schofield, N.A., Leonard, M.E., Gilardoni, L., Finan, T.M. and Poole, P.S. (2001) Use of differential fluorescence induction and optical trapping to isolate environmentally induced genes. Environ Microbiol, 3, 397-406.
4. Meyer, T., Renoud, S., Vigouroux, A., Miomandre, A., Gaillard, V., Kerzaon, I., Prigent-Combaret, C., Comte, G., Moréra, S., Vial, L. et al. (2018) Regulation of hydroxycinnamic acid degradation drives *Agrobacterium fabrum* lifestyles. Mol Plant Microbe Interact, 31, 814-822.

Table S2. Primers used in this study

Target	Primer	Primer sequence (5’-3’)	Fragment length
Transcriptional fusion			
atu1419 promoter	1419FClaI	ATCGATGGATACCATGTCGGCTATCG	583 bp
	1419R	CAATTCGCCACTCAGGATTT	
atu1420 promoter	1420F	TACAAGCATAAAGCTTGCGTCCCCTTCGGCCTT	451 bp
	1420R	GTGGATCCCCCGGGCTTCAACGGGGTTCGGATATTGCT	
***atu1419* and *atu1420* deletion**			
atu1419 upstream region	*atu1419*UpF	GGGCCTTTGCTATTGTGAAA	1034 bp
	*atu1419*UpR	CTGGATTTGCAGCGATTTCCGCCGCTTAGTCGCTGATGAGTGCGA**CATATCTCACCCCTTCCATC**	
atu1419 downstream region	*atu1419*DwF	CTCGCACTCATCAGCGACTAA	1017 bp
	*atu1419*DwR	AGTCGCGATAATCCTTCAGC	
atu1419 deletion verification	*atu1419*ExtF	GCACTGTCCTCAAGCCATCT	3126 bp for WT
	*atu1419*ExtR	CGTCCTTCTTCCAGCCAATA	2445 bp for mutant
atu1420 upstream region	*atu1420*UpF	GTCCTGTCGGTGGTGAAGTT	1018 bp
	*atu1420*UpR	GAACGGGCGCGGGCAGACGGAGCCTTCTCAGGACGCCTTTTTGGT**CATTGTCATCTCCTCCCTGG**	
atu1420 downstream region	*atu1420*DwF	ACCAAAAAGGCGTCCTGA	1024 bp
	*atu1420*DwR	CGACGATGGTTACGATGAAT	
atu1420 deletion verification	*atu1420*ExtF	CGTGCCATCAGGCAGAATA	3959 bp for WT
	*atu1420*ExtR	CAAGGAAAGCGAATGGTTCT	2042 bp for mutant
**Atu1419 expression**			
atu1419 amplification	Atu1419F	GGAATCCATATGAGGCAGGTGGATGCTGC	735 bp
	Atu1419R	GCACTCGAGGTCGCTGATGAGTGCGA	
Gel shift assays			
Patu1416-atu1417	GS 14161417F	ATTCAATTTCTGCGGATAGG	326 bp
	GS 14161417R	CTGTTGAGGCCAACAAGG	

PhcaR	GS hcaRF	ATGTCGATCTGTGCAACC	375 bp
	GS hcaRR	GATATTGACGTCCATCGTCT	
P*atu1418-1419*	GS 14181419F	CCATGTGTCGCAGCATCC	370 bp
	GS 14181419R	CACCGGAATTATCCCAAAAA	
P1- *atu1418-1419*	P1F	AAATATCCAGAAGCTCGGCG	319 bp
	P1R	CATGTTGATGCGATGGTGTC	
P2- *atu1418-1419*	P2F	GACACCATCGCATCAACATG	311 bp
	P2R	CCGTCTATGTGGGCGATGTA	
P3- *atu1418-1419*	P3F	AATCAGGTCTCGCTAAGGGA	320 bp
	P3R	TTCAGCCAGTCCTCCAGC	
P*atu1420* (P1)	GS *atu1420*F	AAGCGCCTGAACTGATTG	268 bp
	GS *atu1420*R	GAACCCCGTTGAAATGCTC	
P*atu1420*s	GS *atu1420*sF	CGTTTCATGTATACATAGATGCGA	144 bp
	GS *atu1420*sR	CGACAACCGGGTATACATACATT	
P*atu1420*s AT	GS *atu1420*sATF	CGTTTCATA**A**TATAT**T**ATAGATGCGA	144 bp
P2- *atu1420*	P2impF	AGGGAGGAGATGACAATGGC	190 bp
	P2impR	GGTGGGACTGGTCAAACAAC	
P1P2- *atu1420*	P1P2F	TCCCTGACGCGGTAATTTTG	263 bp
P3- *atu1420*	P3F	GCACAGGAGCAGCACAAG	247 bp
	P3R	CAGGGAGGGTGTCGACAAAA	
P*virB*	GS virBF	CAAGACATATGTTCGCTGAG	395 bp
	GS virBR	CAGCGGCCCTGTTGCCTTCA	

Table S3. Expression of genes regulated by Atu1419 in the wild-type (WT) strain in presence of citrate. Values refer to fold change at 24 hours obtained by comparison of genes expression in the WT strain without and with 750 µM citrate and correspond to the mean of four technical replicates. The experiment was repeated twice. Empty pOT1e corresponds to plasmid without any promoting region before the *egfp* gene in order to measure basal expression of the system.

**Genes**	**Fold change in the presence / in the absence of citrate**
Patu1418	1.11±0.02
Patu1419	1.08±0.01
Patu1420	1.03±0.08
Empty pOT1e	0.96±0.03

Figure S1. (**A**) Size exclusion chromatograms of Atu1419 (blue line) and upon addition of the palindromic DNA (magenta line) with a tetrameric protein:DNA ratio of 1:2 showing Atu1419 as a tetrameric protein (the theoretical molecular weight of the monomer is ~26542 Da). The oligomeric states were determined by linear regression employing a calibration curve (green line) with Aldolase, Conalbumin and Ovalbumin. The molecular weight of the 10 bp palindrome is estimated to be ~6400 Da. (**B**) SEC-MALS analysis showing the same oligomeric state of Atu1419 as in **A**.

A

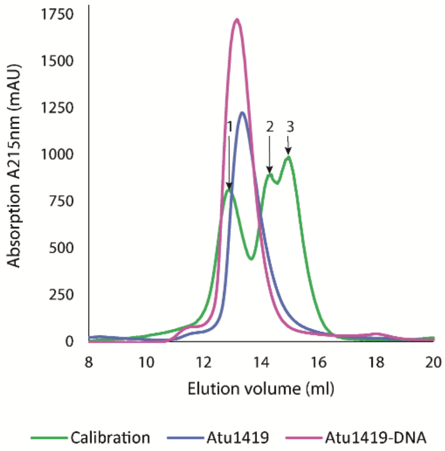

	MW (kDa)	Elution volume (ml)	Estimated MW (kDa)
Calibration			
1- Aldolase	158	12.9	
2- Conalbumin	75	14.36	
3- Ovalbumin	44	14.96	
Atu1419		13.33	113.65
Atu1419-DNA		13.17	122.45

B

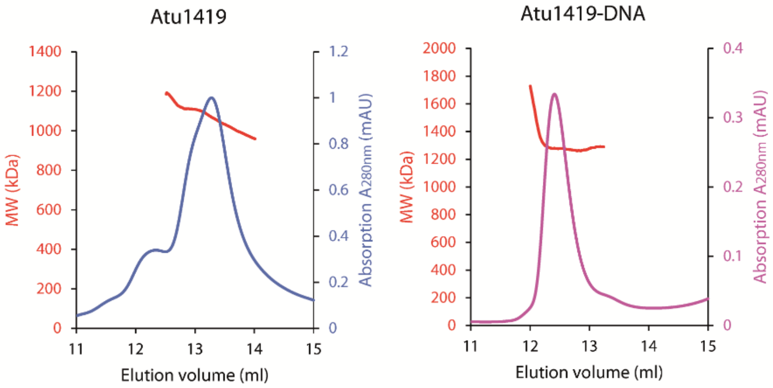

	Elution volume (ml)	Estimated MW (kDa)
Atu1419	13.2	111.11 ± 0.9
Atu1419-DNA	12.4	128 ± 0.4

Figure S2. (**A**) Superposition of the fortuitous citrate ligand (sticks) and Zn^{2+} (ball) in the Atu1419-citrate structures shown in blue for subunit A in the $P2_12_12_1$ structure, in orange for subunit B in the $P2_12_12_1$ structure and in green for subunit A in the $P2_12_12$ structure. (**B**) Interactions between the bound citrate/Zn^{2+} (ball) and subunit A in the $P2_12_12$ structure. Hydrogen bonds are shown as dashed lines in black (distance below 3.2 Å) and metal contacts are shown as dashed lines in red. Residues involved in the interactions are labeled and shown as sticks.

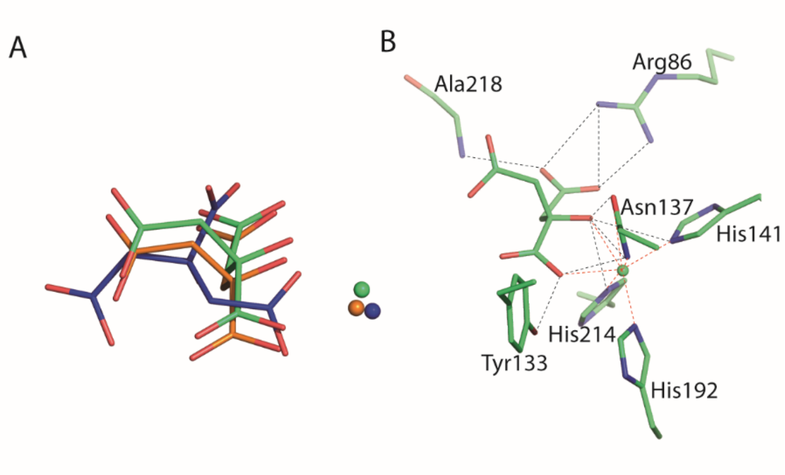

Figure S3. (**A**) Crystal packing of the $P6_422$ structure of Atu1419-DNA complex showing the asymmetric unit subunit in purple and the crystallographic neighbors in grey. The asymmetric unit contains one subunit of Atu1419 shown and one single DNA strand. (**B**) Crystal packing of the $C2_12_12_1$ structure of Atu1419-DNA complex showing the asymmetric unit subunits in magenta and pink and the crystallographic neighbors in gray. The asymmetric unit contains one dimer of Atu1419 and the DNA double strand.

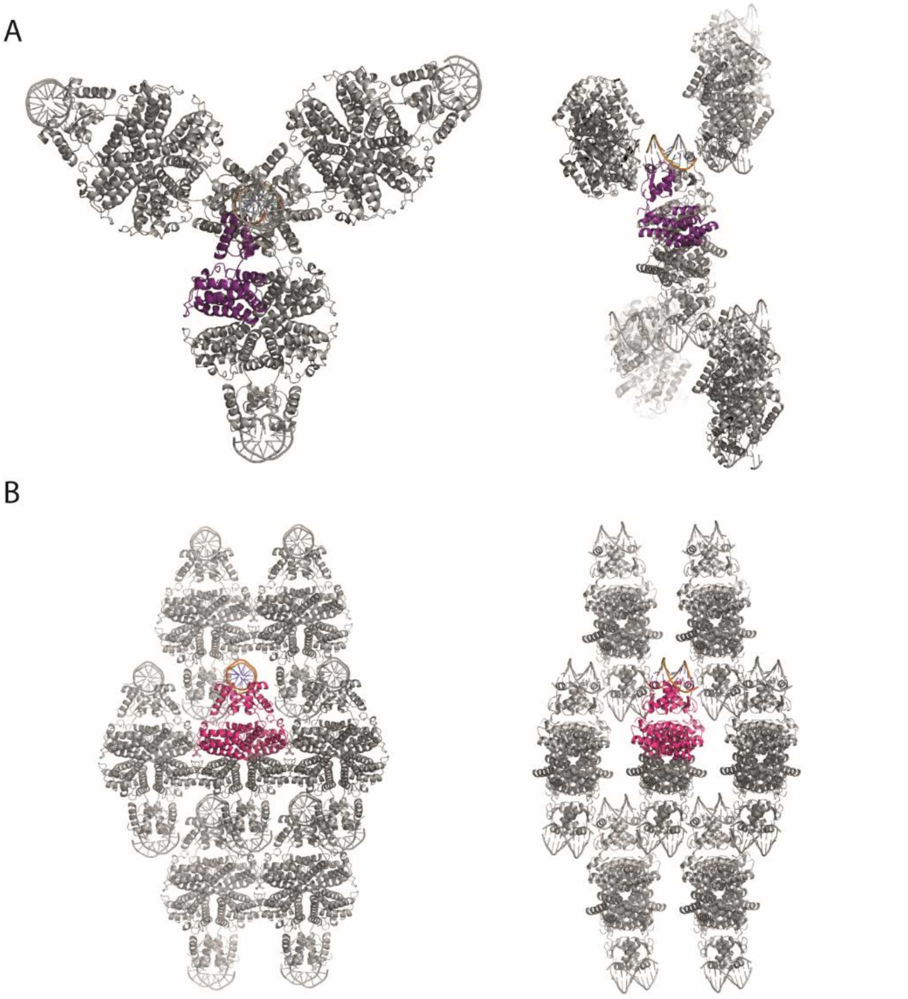

Figure S4. Circular dichroism analysis spectra of wild-type Atu1419 (magenta) and Atu1419-H3A mutant (blue).

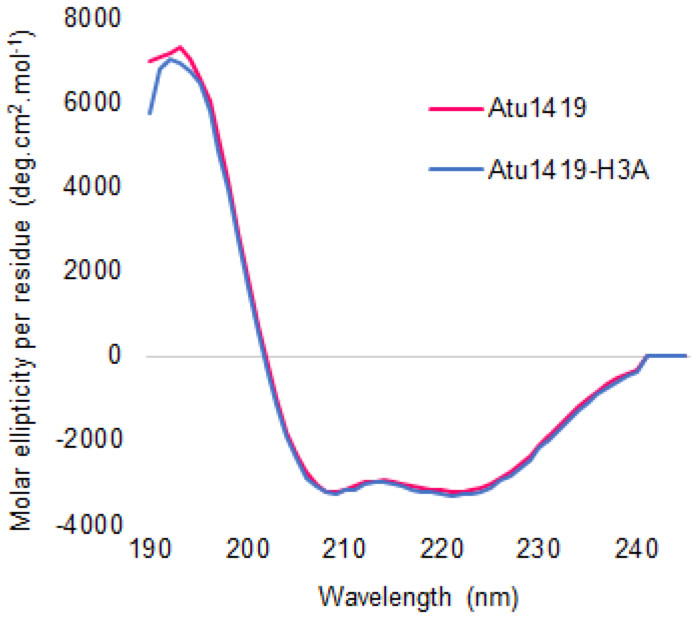

Figure S5. (**A**) Superposition of the subunits A in orange and B in gray of the tetrameric apo Atu1419, with a close-up view showing the comparison of the DNA binding domains of subunits A (orange) and B (gray). (**B**) Comparison of the DNA binding domains of dimers AB between the apoform (in orange) and Atu1419-DNA complex ($P6_422$ structure, purple and gray). The DNA is in green. (**C**) Same comparison as in **B** with Atu1419-citrate complex in $P2_12_12_1$ (cyan) instead of apoform. (**D**) Same comparison as in **B** with Atu1419-citrate complex in $P2_12_12$ (green) instead of apoform.

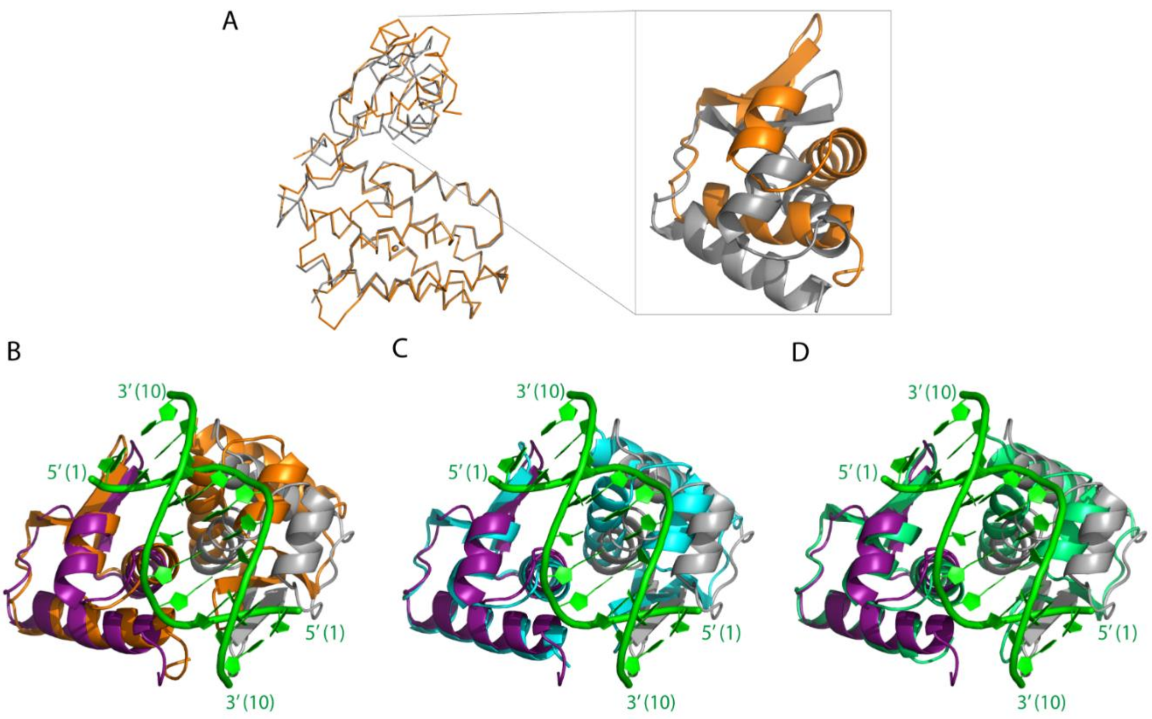

Figure S6. (**A**) Structural comparison of the C-terminal domains of Atu1419 (in orange) and *Thermotoga maritima* TM0439 (PDB 3SXK, in green). Both proteins possess a bound metal ion shown in ball. (**B**) Structural comparison of Atu1419 dimer (in orange) and that of *Escherichia coli* McbR/YncC (PDB 4P9F, in blue), the closest homologue of Atu1419. (**C**) Structural superposition of the conserved histidines in metal binding FCD family members: Atu1419 is shown in orange, *T. maritima* TM0439 (PDB 3SXK) in green, *Pseudomonas syringae* PS5454 (PDB 3C7J) in yellow and *Corynebacterium glutamicum* CGL2915 (PDB ID: 2DI3) in pink. Each respective metal is shown as a sphere in the same color.

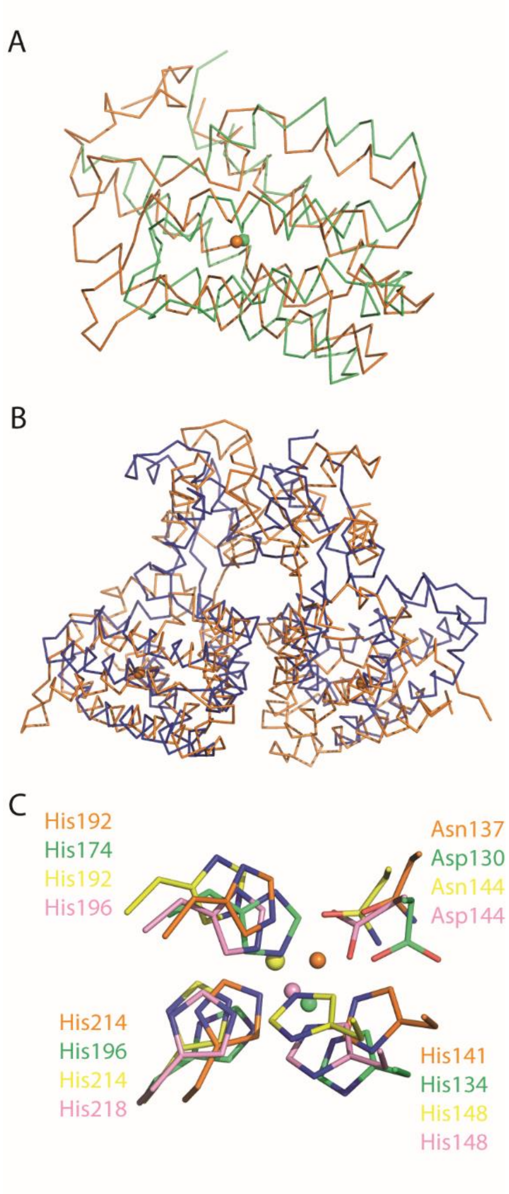

Figure S7. Differential scanning calorimetry thermograms of apo Atu1419 (black) and Atu1419-H3A mutant (blue). The table below indicates the T*m*. DSC experiments were performed twice.

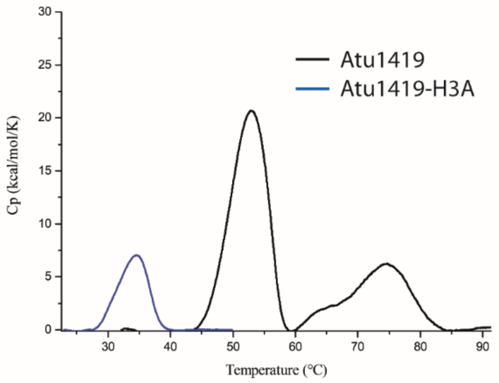

	Tm1 (°C)	ΔH1 (kcal/mol)
**Atu1419**	52.61 ± 0.01	153
**Atu1419-H3A**	34.11 ± 0.01	43

Figure S8. (**A**) Structural comparison of the N-terminal domains bound to DNA of Atu1419 (in magenta) and *E. coli* FadR (PDB 1H9T, in blue) in three views (Left: dimers; Middle: subunits A, Right: subunits B). DNA is orange and green for Atu1419 and FadR, respectively. (**B**) Same comparison with *E.coli* FadR (PDB 1HW2, in pink). (**C**) Same comparison with *V. cholerae* FadR (PDB 4P9U, in gray).

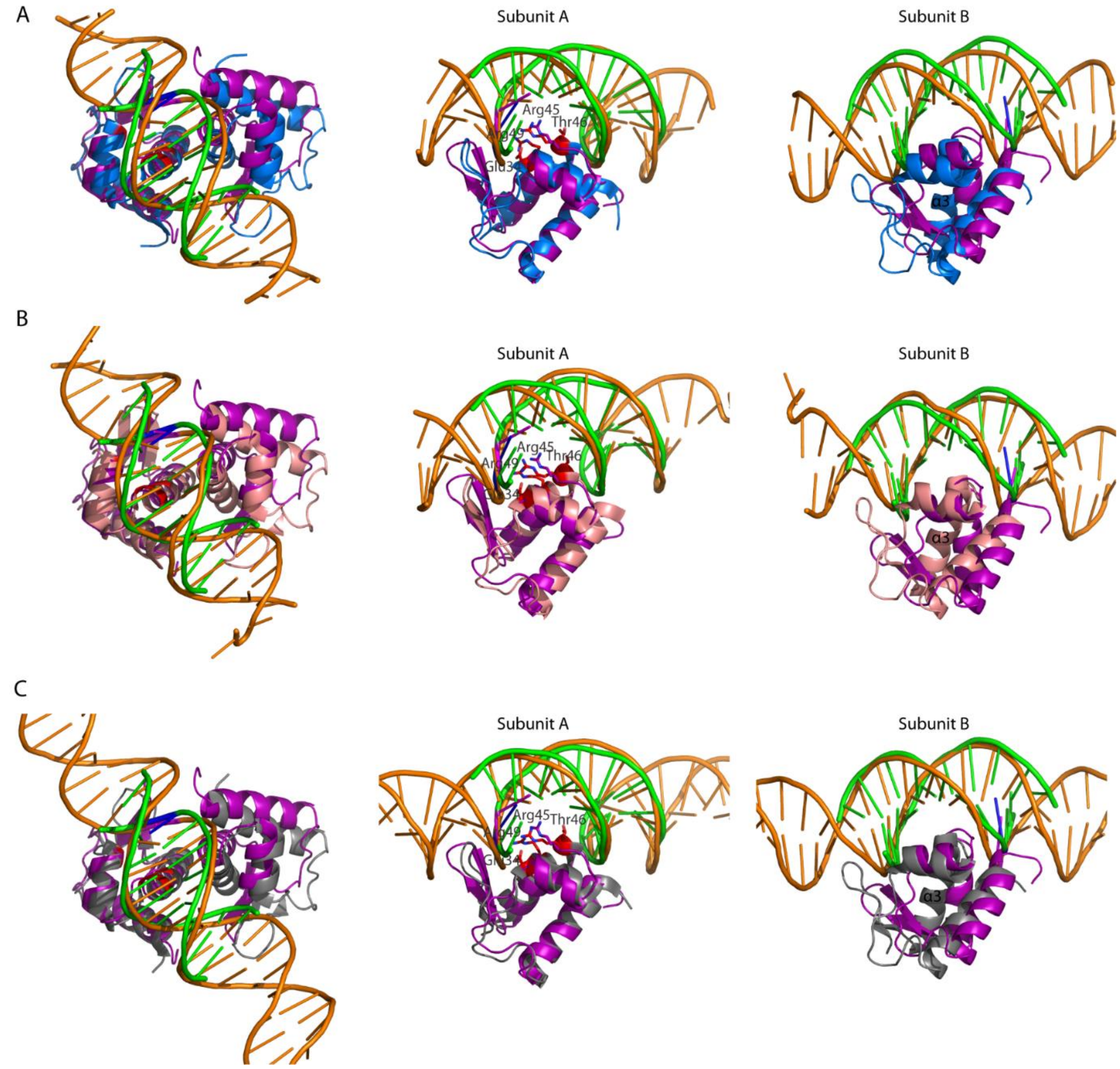

Figure S9. Size exclusion chromatograms of Atu1419 (blue line) and upon addition of MEF (magenta line) with a tetrameric protein:MEF ratio of 1:4 showing Atu1419 as a tetrameric protein (the theoretical molecular weight of the monomer is ~26542 Da). The oligomeric states were determined by linear regression employing a calibration curve with Aldolase, Conalbumin and Ovalbumin as shown in supplementary Figure S1.

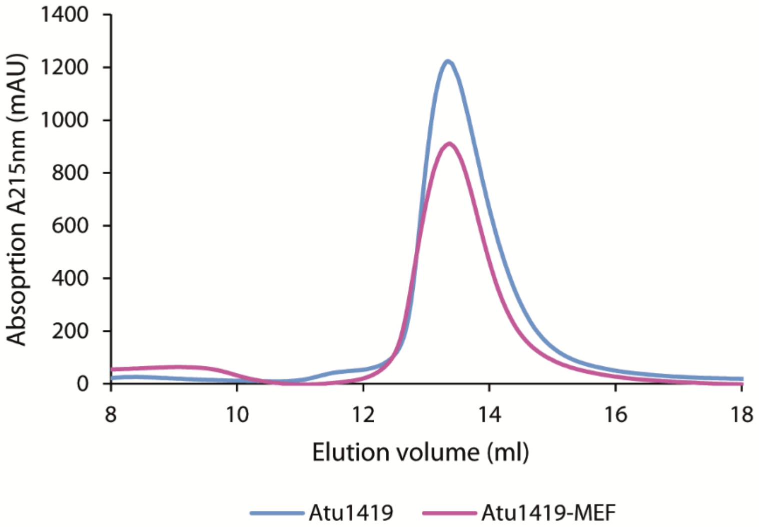

	Elution volume (ml)	Estimated MW (kDa)
Atu1419	13.33	113.65
Atu1419-MEF	13.37	111.55
